# Association between thyroid function and thyroid homeostasis parameters and the prevalence and all-cause and cardiovascular mortality of chronic kidney disease: a population-based study

**DOI:** 10.1186/s12889-025-23695-z

**Published:** 2025-08-09

**Authors:** Xue Liu, Yuhao Zhang, Yuchen Li, Xiude Fan, Haiqing Zhang

**Affiliations:** 1 Department of Endocrinology, Key Laboratory of Endocrine Glucose & Lipids Metabolism and Brain Aging, Ministry of Education, Shandong Provincial Hospital Affiliated to Shandong First Medical University, Jinan, Shandong 250021 China; 2 Department of Urology, Shandong Provincial Hospital, Shandong First Medical University, Jinan, Shandong 250021 China; 3 Shandong Clinical Medical Center of Endocrinology and Metabolism, Jinan, 250021 China; 4 Institute of Endocrinology and Metabolism, Shandong Academy of Clinical Medicine, No. 324, Five-Jing Road, Jinan, Shandong Province 250021 China

**Keywords:** Thyroid function, Thyroid Homeostasis parameters, Chronic Kidney Disease, Mortality, NHANES

## Abstract

**Background:**

To evaluate the relationship between thyroid function and thyroid homeostasis parameters with the prevalence of chronic kidney disease (CKD) and furtherly explore the all-cause and cardiovascular mortality among individuals with CKD using data from the National Health and Nutrition Examination Survey (NHANES) 2007–2012.

**Methods:**

This study included 8,526 adults, including 1,625 patients with CKD. Thyroid function included serum free triiodothyronine (FT3), free thyroxine (FT4) and thyroid-stimulating hormone (TSH). The thyroid homeostasis parameters, including FT3/FT4, thyroid feedback quantile-based index (TFQI_FT4_, TFQI_FT3_), thyrotrophic thyroxine resistance index (TT4RI, TT3RI) and thyroid-stimulating hormone index (TSHI) were calculated. Weighted multivariate logistic regression models to explore the association between thyroid function and thyroid homeostasis parameters and the prevalence of CKD. Cox proportional hazards models were used to investigate the association of thyroid function and thyroid homeostasis parameters with all-cause and cardiovascular mortality among CKD patients. Kaplan–Meier curves compared survival across the quartiles of the thyroid function and thyroid homeostasis parameters among CKD patients. Furthermore, the restricted cubic splines were used to explore the non‑linear relationships.

**Results:**

The weighted multivariate logistic regression models showed that FT4 was positively correlated with the prevalence of CKD, FT3/FT4 and TFQI_FT3_ were negatively correlated with mortality in patients with CKD. The Cox regression models 3 shows that the multivariate-adjusted hazard ratios (HRs) and 95% confidence intervals (CIs) of FT3, FT4 and TSH with the all-cause mortality were 0.66(0.47,0.93), 1.07(1.04,1.10) and 1.01(0.98,1.04). At the same time, FT3/FT4 and TFQI_FT3_ were significantly associated with all-cause mortality after multivariate adjustment. And we further converted thyroid function indicators and thyroid homeostasis parameters from a continuous variable to a categorical variable (quartiles) to conduct the sensitivity analysis. There was no difference in cardiovascular mortality. In crude Kaplan–Meier analyses, there was a U-shaped nonlinear relationship between FT3, TSH, FT3/FT4, TT4RI, TT3RI and TSHI with all-cause mortality, but not FT4, TFQI_FT4_ and TFQI_FT3_. There was an inverted U-shaped relationship between TFQI_FT3_ and TT4RI with cardiovascular mortality, but not FT3, FT4, TSH, FT3/FT4, TFQI_FT4_, TT3RI and TSHI.

**Conclusions:**

Thyroid function and thyroid parameters are closely related to the prevalence of the CKD and all-cause and cardiovascular mortality among individuals with CKD, and the specific mechanisms still required further in-depth research in the future.

**Supplementary Information:**

The online version contains supplementary material available at 10.1186/s12889-025-23695-z.

## Introduction

Thyroxine (T4) serves as the primary secreted and transported variant of thyroid hormone. Its free form can enter cells to undergo deiodination, transforming into free triiodothyronine (FT3). FT3 plays a role in regulating energy metabolism and protein synthesis, while also promoting tissue growth, maturation, and differentiation [1].

The levels of thyroid hormones are controlled by the hypothalamus-pituitary-thyroid (HPT) axis, where the pituitary hormone thyroid-stimulating hormone (TSH) stimulates the production of T4 [2]. Physiologically, the negative feedback loop of the HPT axis mediates an inverse correlation between thyroid hormones and TSH [3, 4]. The central sensitivity to thyroid hormones can be evaluated by composite indices derived from TSH and free thyroxine (FT4). The TSH index (TSHI), thyrotrophic T4 and T3 resistance index (TT4RI, TT3RI) and the newly described novel Thyroid Feedback Quantile-based Index (TFQI_FT4_, TFQI_FT3_) index were well verified for evaluating the central sensitivity to thyroid hormones, and the FT3/FT4 ratio (FT3/FT4) was the index reflecting the peripheral bioavailability of thyroid hormones [5, 6]. FT3/FT4 can provide an estimation of the conversion efficiency of FT4 to FT3, indirectly reflecting the peripheral sensitivity to thyroid hormones. Beyond thyroid function, there has been growing interest in understanding the connection between thyroid hormone sensitivity and metabolic disorders. In recent years, indices of sensitivity to thyroid hormone have been established as dependable predictors of insulin resistance, type 2 diabetes (T2D), cardiometabolic risk, as well as disturbances in glucose and lipid metabolism [5, 7, 8].

Chronic kidney disease (CKD) is characterized by anomalies in kidney structure or function that persist for more than 3 months and have health implications [9]. The burden of CKD on public health is increasing in severity [10]. As the awareness of its status as a significant global health concern continues to rise, there has been an increasing focus on modifiable factors that influence the mortality associated with CKD. The diagnostic criteria for CKD align with the guidelines provided in the Kidney Disease: Improving Global Outcomes (KDIGO) 2021 Clinical Practice Guideline for the Management of Blood Pressure in Chronic Kidney Disease.

A growing body of data indicates that different disturbances in thyroid functional tests might be linked to mortality in individuals with CKD [11, 12]. The Mendelian randomization study by Ellervik et al. indicated a directional association between hypothyroidism, elevated TSH, TPO antibodies, and increased risk of CKD [13]. Additionally, a prospective cohort study involving 104,633 individuals with normal thyroid hormone levels and no history of thyroid disease revealed that high TSH levels and low FT3 levels are associated with an increased risk of incident CKD [14]. Furthermore, a cross-sectional study involving 1,571 participants found that elevated serum thyroid function parameters are correlated with increased prevalence of CKD in the elderly, independent of the effects of age, diabetes, and hypertension [15]. Apart from FT3, FT4, and TSH, composite indices offer a more comprehensive representation of thyroid homeostasis, owing to the intricate interplay among FT3, FT4, and TSH [3, 16, 17]. However, few studies have focused on the relationship between thyroid homeostasis index and CKD.

Our study systematically evaluated the relationship between thyroid function and homeostasis parameters with the prevalence of the CKD and all-cause and cardiovascular mortality among individuals with CKD using data from the National Health and Nutrition Examination Survey (NHANES) 2007–2012, which will provide a more specific thyroid management strategy for CKD patients.

## Methods

### Study population

**Fig. 1:**
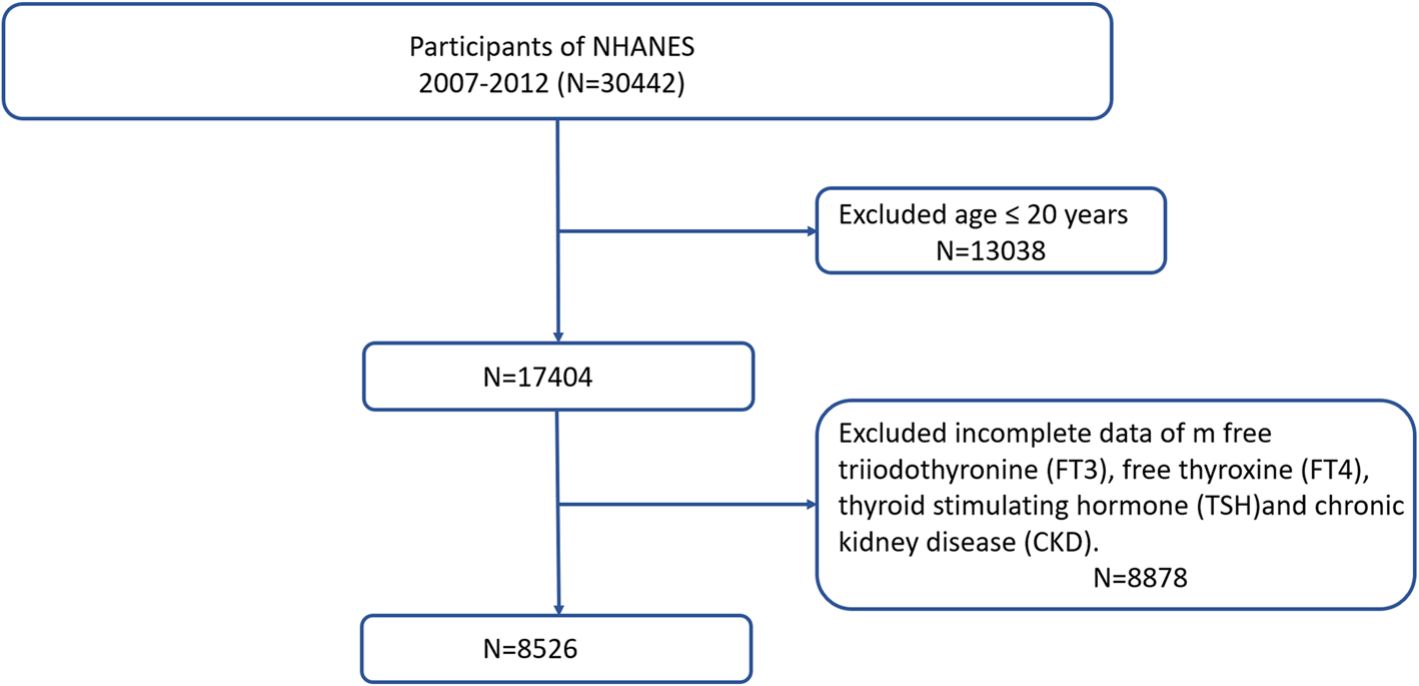
Study flowchart. National Health and Nutrition Examination Survey (NHANES), 2007- 2012

NHANES, an ongoing and recurrent study carried out by the US National Center for Health Statistics (NCHS), encompasses a nationwide database that encompasses data concerning the health and nutritional well-being of both adults and children in the United States. We merged three cycles of NHANES data from 2007 to 2012 for this research (*n* = 30,442). After a series of screenings, we finally selected out of 8,526 participants for the final data analysis. Then, we screen participants according to the exclusion criteria listed below: (1) participants aged ≤ 20 years (*n* = 13,058); (2) participants without FT4, FT3, TSH, eGFR and UACR (*n* = 8,858). Figure 1 depicts the full data integration process. Protocols used in the NHANES were approved by US National Center for Health Statistics Research Ethics Review Board, and written informed consent was provided by all participants. NHANES provides detailed information over the web (www.cdc.gov/nchs/nhanes/index.htm).

### Determination of serum thyroid function

Serum FT3 and FT4 were measured using a competitive binding immunoenzymatic assay and a two-step enzyme immunoassay, respectively. TSH levels were quantified utilizing the Access HYPERsensitive hTSH Assay, employing a two-site immunoenzymatic ("sandwich") method. The reference range for thyroid hormone levels in NHANES 2007–2012 included a serum FT4 level of 0.6–1.6 ng/dL, a serum FT3 level of 2.5–3.9 ng/dL, and a TSH level of 0.34–5.60 mIU/L.

Peripheral index of thyroid hormone sensitivity was calculated as: FT3/FT4 = FT3 (pmol/L)/FT4 (pmol/L) [18]. Higher values indicated higher peripheral sensitivity to thyroid hormones. The indices of thyroid hormone central sensitivity were calculated with following formulas: TSHI was calculated as ln TSH (mIU/L) + 0.1345*FT4 (pmol/L) [19].TT4RI was calculated as FT4 (pmol/L)* TSH (mIU/L) [20].TT3RI was calculated as FT3 (pmol/L)* TSH (mIU/L). For TSHI, TT4RI and TT3RI, higher values indicated lower central sensitivity to thyroid hormones [17]. Thyroid Feedback Quantile-based Index (TFQI) is achieved by applying the population empirical cumulative distribution function (cdf) to hormone concentration [5]. TFQI_FT4_ was calculated as cdf FT4– (1– cdf TSH). TFQI_FT3_ was calculated as cdf FT3– (1– cdf TSH). The TFQI values are between − 1 and 1, with negative and positive values indicating good and poor sensitivity to FT4 or FT3, respectively [21].

### Ascertainment of mortality outcomes

NHANES linked mortality public use files are available for continuous NHANES periods 2007 to 2008, 2009 to 2010 and 2011–2012. Follow-up time is from medical examination until December 31, 2019. The mortality data from the National Center for Health Statistics were established through a probabilistic record matching process that linked NHANES participants with National Death Index (NDI) death certificate information. Mortality status is determined through a probabilistic matching procedure that links NHANES data with NDI death certificate records.

### Assessment of covariates

Demographic, clinical interview, physical examination, and additional laboratory variables were collected following the procedures outlined in NHANES operation manuals [22].

Continuous variables consisted of age (years), body mass index (BMI, kg/m^2^), systolic blood pressure (SBP, mmHg), diastolic blood pressure (DBP, mmHg), alanine aminotransferase (ALT, U/L), aspartate aminotransferase (AST, U/L), iodine, urine (ug/L), eGFR (mL/min/1.73 m^2^) and UACR (mg/g). The systolic and diastolic blood pressure values were derived from the average of four blood pressure measurements. eGFR was calculated according to the CKD-EPI (CKD Epidemiology Collaboration) equation, using calibrated creatinine level [23]. UACR was calculated by dividing the urinary albumin concentration in milligrams by the urinary creatinine concentration in grams [24].

Categorical variables included sex (male, female), educational level (or level of education) (Under high school, High school or equivalent, College graduate or above), and any hyperlipidemia. Within the primary sample dataset, diabetes mellitus (DM) was characterized by a fasting plasma glucose level of ≥ 7.0 mmol/L, a glycohemoglobin level of ≥ 6.5%, the utilization of diabetes medication or insulin, or a self-reported diagnosis of diabetes [25], which was divided into four status: no, impaired glucose tolerance (IGT), impaired fasting glucose (IFG), and DM. Triglyceride ≥ 150 mg/dl, total cholesterol ≥ 200 mg/dl, low density lipoprotein (LDL-C) ≥ 130 mg/dl, high density lipoprotein (HDL-C) < 40 mg/dl for male and < 50 mg/dl for female and taking anti-hyperlipidemic drugs were analyzed, as they can be considered as hyperlipidemia.

### Statistical analysis

All analyses accounted for the complex survey design using appropriate survey weights. Weighted analysis was conducted using the Survey package in R.

Data were expressed as mean ± standard deviation (SD) for continuous variables and as numbers (proportions) for categorical variables. The chi-squared test was applied to categorical variables, while one-way ANOVA was used for normally distributed continuous variables, and the Kruskal–Wallis test was used for skewed continuous variables. Weighted multivariate logistic regression models were performed to estimate the OR and 95% confidence interval associated with the prevalence of CKD. In model 1, no variable was adjusted for. While in model 2, demographic data (age, sex, education level and race) were adjusted for; and SBP, DBP, BMI, AST, ALT, Urine iodine, DM and Hyperlipidemia were further adjusted in model 3. Cox proportional hazards regression estimated hazard ratios (HRs) and 95% CIs of all-cause and cardiovascular mortality among individuals with CKD for each SD increment of the thyroid function and thyroid parameters. In model 1, no variable was adjusted for. While in model 2, demographic data (age, sex, education level and race) were adjusted for; and SBP, DBP, BMI, AST, ALT, Urine iodine, eGFR, UACR, DM and Hyperlipidemia were further adjusted in model 3. Kaplan–Meier curves visually compared survival across the quartiles of the thyroid parameters. Furthermore, after adjusted the age, sex, education level, SBP, DBP, BMI, AST, ALT, Urine iodine, eGFR, UACR, DM and Hyperlipidemia, the restricted cubic splines were used to explore the non‑linear relationships between thyroid function and thyroid homeostasis parameters and all-cause and cardiovascular mortality among individuals with CKD.

To address missing covariate data, we employed multiple multivariate imputations. Our aim was to optimize statistical power and mitigate potential bias that could arise if covariates with missing data were excluded from the analyses. We generated five imputed datasets using chained equations through the Mice package in R statistical software [26]. *P*-value < 0.05 was considered statistically significant. All analyses were performed with R version 4.3.1.

## Results

### Participant characteristics

In our study, 8526 participants represented the U.S. population 20 years.

of age and older participated in the analysis, including 1,625 patients with CKD. By the follow-up deadline, in CKD patients, 663 patients had died, of which 181 died from cardiovascular diseases. Compared with the non-CKD group, CKD patients had lower FT3, FT3/FT4, TFQI_FT3_ and higher FT4, TFQI_FT4_, TT4RI and TSHI (Table 1). And there was statistically significant difference among the CKD and non-CKD in terms of age, sex, race, education levels, thyroglobulin (Tg), BMI, SBP, DBP, Urine iodine-group, hyperlipidemia or not, DM or not (Table 1). We compared the baseline characteristics and inter-group differences of thyroid hormones and thyroid parameters among different age groups, sex, and race in CKD patients (Supplementary Table 1). In CKD patients, older adults and females have higher FT4 levels and lower FT3 levels. Additionally, older adults exhibit relatively higher TSH levels. Indices of resistance to FT4 were higher among older people and indices of resistance to FT3 were higher among younger people (Supplementary Table 1). Meanwhile, we analyzed clinical characteristics of subjects by quartiles of FT3/FT4, TFQI_FT4_, TFQI_FT3_, TT4RI, TT3RI and TSHI (Supplementary Table 2–10) Following KDIGO guidelines, we classified CKD patients into four distinct risk categories: low (G1A1-G2A1), moderate (G1A2-G3aA1), high (G3aA2-G3bA3), and very high risk (G4A1-G5A3) based on combined eGFR and albuminuria levels.We also compared baseline characteristics across these four risk strata (Supplementary Table 11).

**Table 1:** Baseline characteristics of included participants in NHANES 2007–2012

Variable	No-CKD	CKD	*P* value
Age (years)	45.4 ± 0.36	61.94 ± 0.71	< 0.0001*
Age-group (years)			< 0.0001*
>= 60	1868(19.50)	1116(61.61)	
18–39	2574(38.41)	163(14.03)	
40–59	2459(42.09)	346(24.36)	
Sex			< 0.001*
female	3468(50.57)	853(57.17)	
male	3433(49.43)	772(42.83)	
Race			< 0.001*
mexican american	1162(8.25)	219(7.03)	
non-hispanic black	1301(9.96)	364(13.49)	
non-hispanic white	3131(68.58)	811(69.56)	
other hispanic	793(5.90)	146(4.97)	
other race	514(7.31)	85(4.96)	
Education levels			< 0.0001*
College graduate or above	3442(61.02)	629(46.48)	
High school or equivalent	2708(33.59)	668(40.11)	
Under high school	748(5.40)	323(13.41)	
Tg (ng/mL)	15.44 ± 0.56	18.76 ± 0.88	0.003*
TgAb (IU/mL)	8.95 ± 1.30	16.45 ± 4.19	0.09
TPOAb (IU/mL)	23.81 ± 1.88	23.58 ± 3.23	0.95
BMI (kg/m^2^)	28.46 ± 0.13	30.07 ± 0.27	< 0.0001*
ALT (U/L)	26.25 ± 0.39	24.65 ± 0.70	0.05*
AST (U/L)	26.17 ± 0.25	27.88 ± 1.09	0.13
SBP (mmHg)	119.20 ± 0.37	131.05 ± 0.62	< 0.0001*
DBP (mmHg)	70.83 ± 0.33	68.74 ± 0.53	< 0.0001*
eGFR(mL/min/1.73m^2^)	98.3 ± 0.50	71.74 ± 1.11	< 0.0001*
uACR (mg/g)	7.66 ± 0.13	178.69 ± 17.09	< 0.0001*
Urine iodine (ug/L)	302.00 ± 67.05	371.64 ± 41.93	0.39
Urine iodine-group			0.004*
≤ 100	2236(34.54)	466(29.51)	
100–199	2245(32.03)	508(31.01)	
> 199	2402(33.43)	648(39.48)	
Hyperlipidemia			< 0.0001*
No	1892(28.01)	262(16.14)	
Yes	5009(71.99)	1363(83.86)	
DM			< 0.0001*
No	989(10.93)	675(35.05)	
IGT	313(3.87)	78(4.93)	
IFG	319(4.53)	71(4.08)	
Yes	5200(80.66)	797(55.95)	
FT3 (pg/mL)	3.19 ± 0.01	3.01 ± 0.02	< 0.0001*
FT4 (pmol/L)	10.30 ± 0.07	11.03 ± 0.11	< 0.0001*
TSH (mIU/L)	2.05 ± 0.07	2.17 ± 0.06	0.19
FT3/FT4	0.49 ± 0.00	0.44 ± 0.00	< 0.0001*
TFQI_FT4_	0.05 ± 0.01	0.16 ± 0.02	< 0.0001*
TFQI_FT3_	0.04 ± 0.01	−0.06 ± 0.02	< 0.0001*
TT4RI	19.93 ± 0.47	22.61 ± 0.64	< 0.001*
TT3RI	9.83 ± 0.33	9.81 ± 0.25	0.95
TSHI	1.81 ± 0.02	1.96 ± 0.03	< 0.0001*

Data were presented as mean ± SD or median (interquartile ranges) for continuous variables, and numbers (proportions) for categorical variables

Tg thyroglobulin, TgAb thyroglobulin antibody, TPOAb thyroid peroxidase antibody, BMI body mass index, ALT glutamic-pyruvic transaminase, AST glutamic oxaloacetic transaminase, SBP systolic pressure, DBP diastolic pressure, eGFR estimated glomerular filtration rate, UACR urinary albumin to creatinine ratio, DM diabetes mellitus, IGT impaired glucose tolerance, IFG impaired fasting glucose, FT3 triiodothyronine, FT4 free thyroxine, TSH thyroid-stimulating hormone, TSHI TSH index, TT4RI thyrotrophic T4 resistance index, TT3RI thyrotrophic T3 resistance index, TFQI_FT4_, TFQI_FT3_ thyroid Feedback Quantile-based Index, FT3/FT4 FT3/FT4 ratio

^*^p < 0.05

### Correlations of thyroid function and thyroid homeostasis parameters with the prevalence of CKD

We conducted weighted multivariate logistic regression models to explore the association between thyroid function and thyroid homeostasis parameters and CKD (Table 2). After adjusted the age, sex, education level, race, SBP, DBP, BMI, AST, ALT, Urine iodine, DM and Hyperlipidemia (Model 3), FT4 was positively correlated with the prevalence of CKD (OR = 1.08; 95% CI = 1.05–1.12, *P* < 0.0001).FT3/FT4 and TFQI_FT3_ were negatively correlated with mortality in patients with CKD (OR = 0.13; 95% CI = 0.05–0.32, *P* < 0.0001; OR = 0.76; 95% CI = 0.58–1.00, *P* = 0.05).

**Table 2:** Correlations of thyroid function and thyroid homeostasis parameters with the prevalence of CKD

	**Model 1**		**Model 2**		**Model 3**	
	OR (95% CI)	*P* value	OR (95% CI)	*P* value	OR (95% CI)	*P* value
FT3	0.28(0.22,0.36)	< 0.0001*	0.82(0.63,1.08)	0.16	0.80(0.61,1.06)	0.11
FT4	1.15(1.11,1.19)	< 0.0001*	1.08(1.05,1.12)	< 0.0001*	1.08(1.05,1.12)	< 0.0001*
TSH	1.01(0.99,1.02)	0.25	1.00(0.98,1.01)	0.78	0.99(0.97,1.01)	0.30
FT3/FT4	0.00(0.00,0.01)	< 0.0001*	0.14(0.05,0.36)	< 0.001*	0.13(0.05,0.32)	< 0.0001*
TFQI_FT4_	2.26(1.67,3.04)	< 0.0001*	1.40(1.01,1.94)	0.04*	1.31(0.95,1.81)	0.09
TFQI_FT3_	0.50(0.40,0.63)	< 0.0001*	0.83(0.61,1.12)	0.21	0.76(0.58,1.00)	0.05*
TT4RI	1.00(1.00,1.01)	0.003*	1.00(1.00,1.00)	0.51	1.00(1.00,1.00)	0.98
TT3RI	1.00(1.00,1.00)	0.95	1.00(0.99,1.00)	0.58	1.00(0.99,1.00)	0.24
TSHI	1.34(1.17,1.54)	< 0.0001*	1.09(0.94,1.27)	0.26	1.04(0.90,1.21)	0.58

Model 1: Non-adjusted

Model 2: Adjusted for age, sex, education level and race

Model 3: Adjusted for age, sex, education level, race, SBP, DBP, BMI, ALT, AST, urine iodine, DM, Hyperlipidemia

FT3 triiodothyronine, FT4 free thyroxine, TSH thyroid-stimulating hormone, TSHI TSH index, TT4RI thyrotrophic T4 resistance index, TT3RI thyrotrophic T3 resistance index, TFQIFT4, TFQIFT3 thyroid Feedback Quantile-based Index, FT3/FT4 FT3/FT4 ratio

^*^p < 0.05

### Correlations of thyroid function with the all-cause mortality and cardiovascular mortality among individuals with CKD

**Fig. 2:**
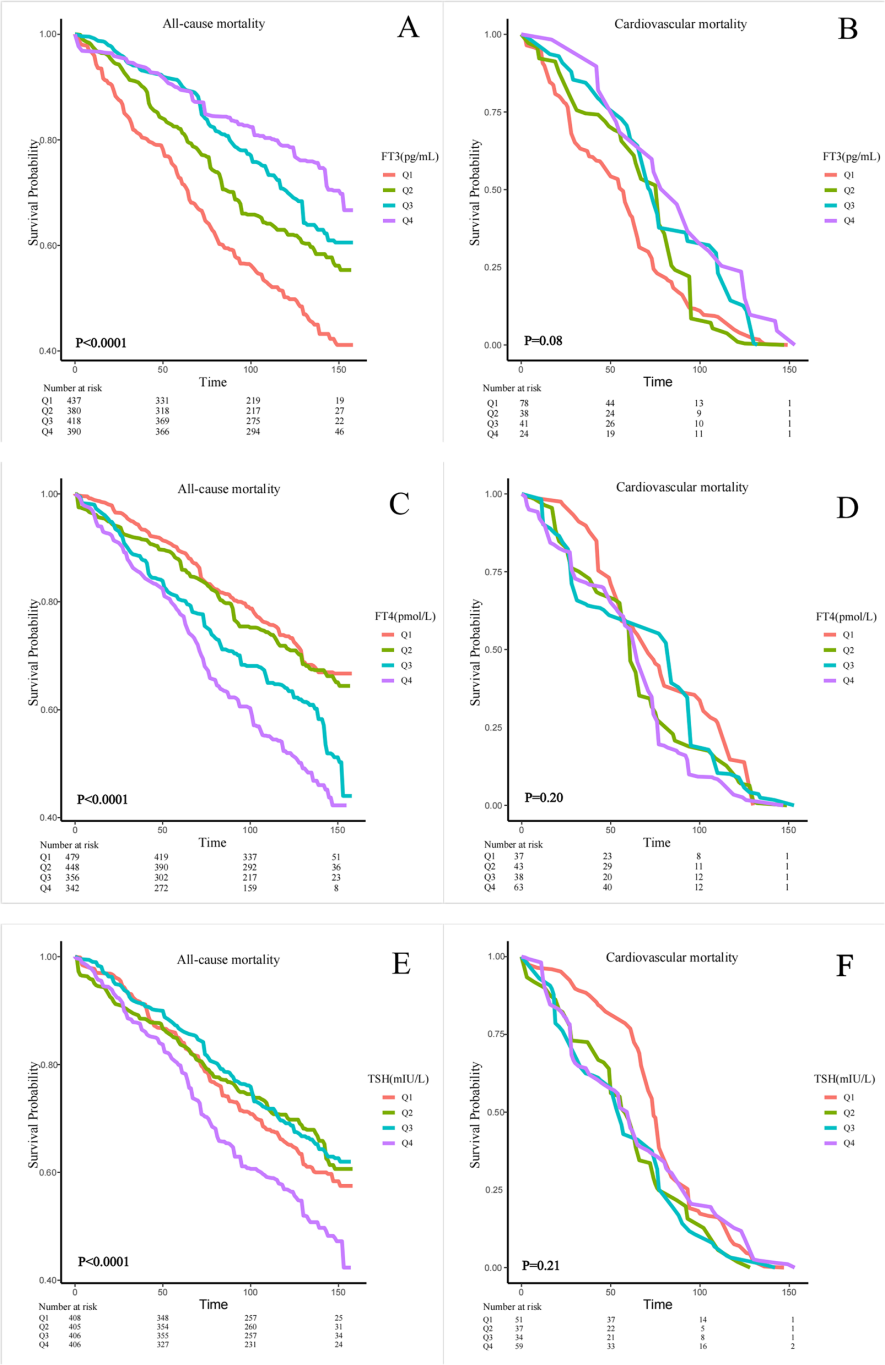
Kaplan–Meier survival estimates all-cause and cardiovascular mortality across the quartiles of the thyroid function (FT3, FT4, TSH) among individuals with CKD

**Fig. 3:**
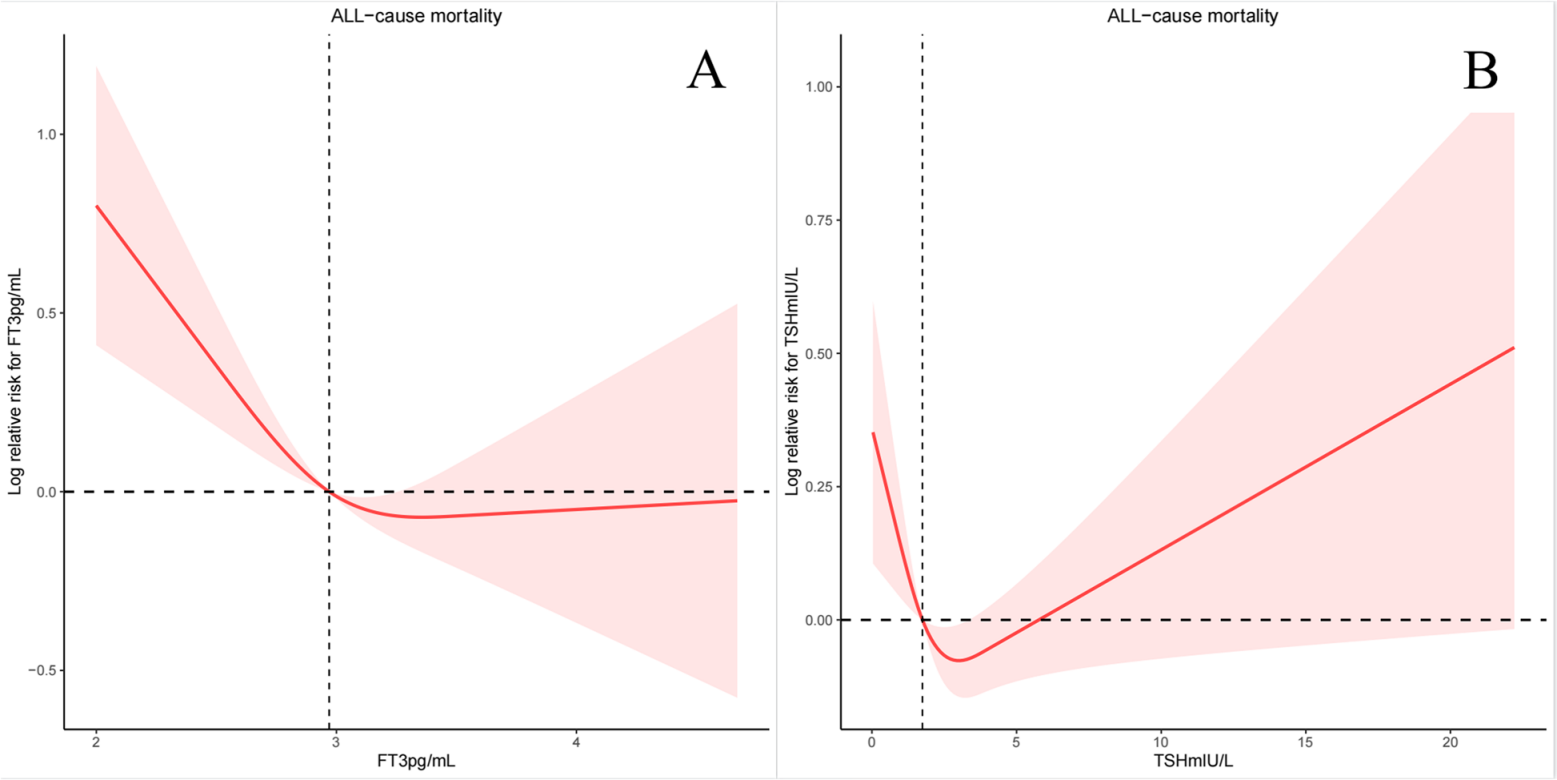
Association between FT3 and TSH with All-Cause Mortality among individuals with CKD using a restricted cubic spline regression model

We designed 3 Cox regression models to investigate the independent role of FT3, FT4 and TSH in mortality. After multivariate adjustment including age, sex, race/ethnicity, education level, SBP, DBP, ALT, AST, BMI, history of hyperlipidemia or diabetes (Model 3), FT3, FT4 and TSH were significantly associated with all-cause mortality. The multivariate-adjusted HR and 95% confidence intervals (CIs) of FT3, FT4 and TSH with the all-cause mortality were 0.66(0.47,0.93), 1.07(1.04,1.10) and 1.01(0.98,1.04). We further converted FT3, FT4 and TSH from a continuous variable to a categorical variable (quartiles) to conduct the sensitivity analysis. The HRs and 95% CIs from lowest to highest FT3 categories were 1.00 (reference), (HR = 0.70; 95% CI = 0.51–0.96, *P* = 0.02), (HR = 0.68; 95% CI = 0.50–0.93, *P* = 0.01), and (HR = 0.78; 95% CI = 0.53–1.15, *P* = 0.21), respectively, for all-cause mortality (P trend = 0.079). The HRs and 95% CIs from lowest to highest FT4 categories were 1.00 (reference), (HR = 0.89; 95% CI = 0.63–1.27, *P* = 0.52), (HR = 1.20; 95% CI = 0.79–1.82, *P* = 0.39), and (HR = 1.41; 95% CI = 1.04–1.93, *P* = 0.03), respectively, for all-cause mortality (P trend = 0.007) (Table 3). However, we found no difference in cardiovascular mortality (Table 3). In crude Kaplan–Meier analyses, FT3, FT4 and TSH were also associated with all-cause mortality in CKD patients (Fig. 2A, C, E), but not associated with cardiovascular mortality (Fig. 2B, D, F). At the same time, we also conducted a sensitivity analysis, and after stratifying by age to analyze the K-M analyses, we found that the differences present in the K-M analysis were more significant in the population aged 60 and above (Supplementary Fig. 1). There was a nonlinear relationship between FT3 and TSH with all-cause mortality (P for overall = 0.0002, P for nonlinear = 0.0049, Fig. 3A; P for overall = 0.0059, P for nonlinear = 0.0025, Fig. 3B, respectively), but not FT4 (P for overall = 0, P for nonlinear = 0.3436). The changepoints between FT3 and TSH with all-cause mortality were 3.355 pg/mL and 3.044 mIU/L, respectively. There was no nonlinear relationship between FT3, FT4 and TSH with cardiovascular mortality (all P for overall > 0.05, P for nonlinear > 0.05). We then examined the association between thyroid function within each risk group with all-cause and cardiovascular mortality in each stratum (Supplementary Table 12–13).

**Table 3:** Correlations of thyroid function with the all-cause mortality and cardiovascular mortality among individuals with CKD

	**All-cause mortality**								**cardiovascular mortality**							
	Model 1		Model 2			Model 3			Model 1		Model 2			Model 3		
	HR (95% CI)	P		HR (95% CI)	P		HR (95% CI)	P	HR (95% CI)	P		HR (95% CI)	P		HR (95% CI)	P
FT3																
Continuous	0.33(0.23,0.48)	< 0.0001		0.70(0.48,1.02)	0.06		0.66(0.47,0.93)	0.02	0.43(0.24,0.75)	0.003		0.49(0.29,0.83)	0.01		0.57(0.34,0.95)	0.03
Quartile 1	Ref	Ref		Ref	Ref		Ref	Ref	Ref	Ref		Ref	Ref		Ref	Ref
Quartile 2	0.67(0.49,0.91)	0.01		0.70(0.52,0.95)	0.02		0.70(0.51,0.96)	0.02	0.82(0.49,1.38)	0.45		0.95(0.57,1.61)	0.86		1.13(0.63,2.04)	
Quartile 3	0.49(0.40,0.61)	< 0.0001		0.68(0.51,0.90)	0.01		0.68(0.50,0.93)	0.01	0.60(0.37,0.95)	0.03		0.63(0.37,1.07)	0.09		0.75(0.43,1.32)	
Quartile 4	0.36(0.25,0.51)	< 0.0001		0.82(0.55,1.22)	0.32		0.78(0.53,1.15)	0.21	0.45(0.25,0.81)	0.01		0.47(0.25,0.89)	0.02		0.53(0.26,1.05)	
P for trend		< 0.0001			< 0.0001			0.079		0.004			0.032			0.095
FT4																
Continuous	1.06(1.03,1.10)	< 0.0001		1.07(1.04,1.10)	< 0.0001		1.07(1.04,1.10)	< 0.0001	1.05(1.00,1.10)	0.06		1.03(0.98,1.09)	0.22		1.03(0.97,1.10)	0.27
Quartile 1	Ref	Ref		Ref	Ref		Ref	Ref	Ref	Ref		Ref	Ref		Ref	Ref
Quartile 2	1.09(0.76,1.57)	0.65		0.89(0.63,1.26)	0.51		0.89(0.63,1.27)	0.52	1.37(0.71,2.66)	0.35		1.06(0.61,1.84)	0.83		1.31(0.71,2.41)	0.39
Quartile 3	1.53(0.99,2.35)	0.05		1.32(0.86,2.01)	0.20		1.20(0.79,1.82)	0.39	1.10(0.60,2.01)	0.77		1.40(0.86,2.27)	0.17		1.48(0.89,2.44)	0.13
Quartile 4	2.10(1.47,3.01)	< 0.0001		1.46(1.08,1.99)	0.01		1.41(1.04,1.93)	0.03	1.62(1.04,2.52)	0.03		1.22(0.81,1.85)	0.35		1.25(0.78,2.02)	0.35
P for trend		< 0.0001			0.002			0.007		0.042			0.228			0.456
TSH																
Continuous	1.03(1.01,1.05)	0.01		1.01(0.99,1.04)	0.30		1.01(0.98,1.04)	0.45	0.99(0.86,1.14)	0.86		1.03(0.94,1.14)	0.49		1.00(0.85,1.17)	0.97
Quartile 1	Ref	Ref		Ref	Ref		Ref	Ref	Ref	Ref		Ref	Ref		Ref	Ref
Quartile 2	0.87(0.62,1.22)	0.42		0.83(0.59,1.18)	0.31		0.85(0.60,1.21)	0.37	1.48(1.00,2.20)	0.05		1.97(1.21,3.19)	0.01		2.01(1.21,3.34)	0.01
Quartile 3	0.85(0.62,1.15)	0.29		0.69(0.50,0.94)	0.02		0.67(0.50,0.89)	0.01	1.45(0.97,2.15)	0.07		1.57(0.97,2.54)	0.07		1.55(0.84,2.87)	0.16
Quartile 4	1.38(1.06,1.81)	0.02		0.96(0.74,1.26)	0.79		0.91(0.71,1.18)	0.49	1.08(0.56,2.07)	0.83		1.51(0.83,2.75)	0.18		1.34(0.62,2.89)	0.46
P for trend		0.037			0.56			0.256		0.849			0.183			0.446

Model 1: Non-adjusted

Model 2: Adjusted for age, sex, education level, race, SBP and DBP

Model 3: Adjusted for age, sex, education level, race, SBP, DBP, BMI, ALT, AST, urine iodine, DM, Hyperlipidemia, eGFR and UACR

### Correlations of thyroid homeostasis parameters with the all-cause mortality and cardiovascular mortality among individuals with CKD

**Fig. 4:**
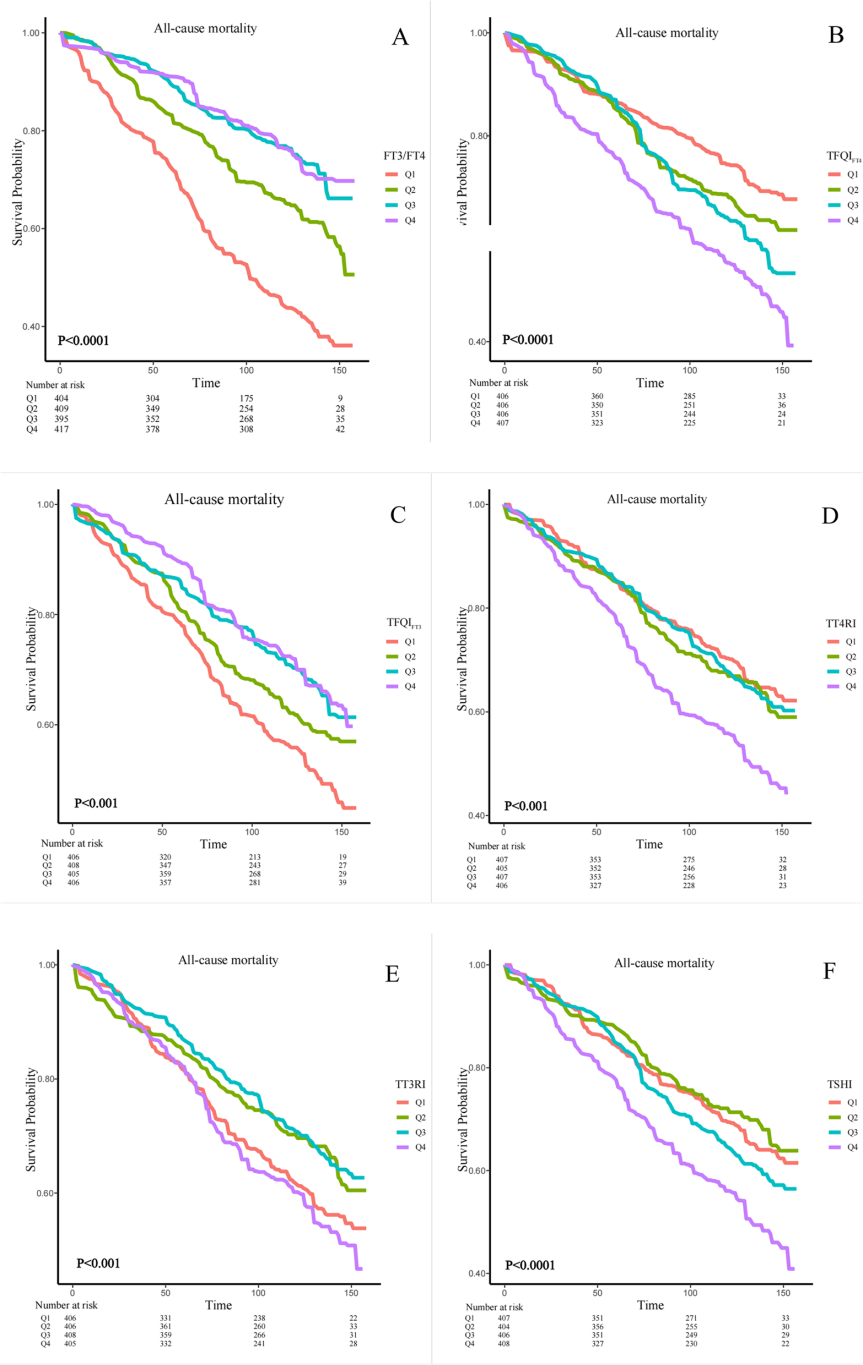
Kaplan–Meier survival estimates all-cause mortality across the quartiles of the thyroid homeostasis parameters (FT3/FT4, TFQI_FT4_, TFQI_FT3_, TT4RI, TT3RI, TSHI) among individuals with CKD

**Fig. 5:**
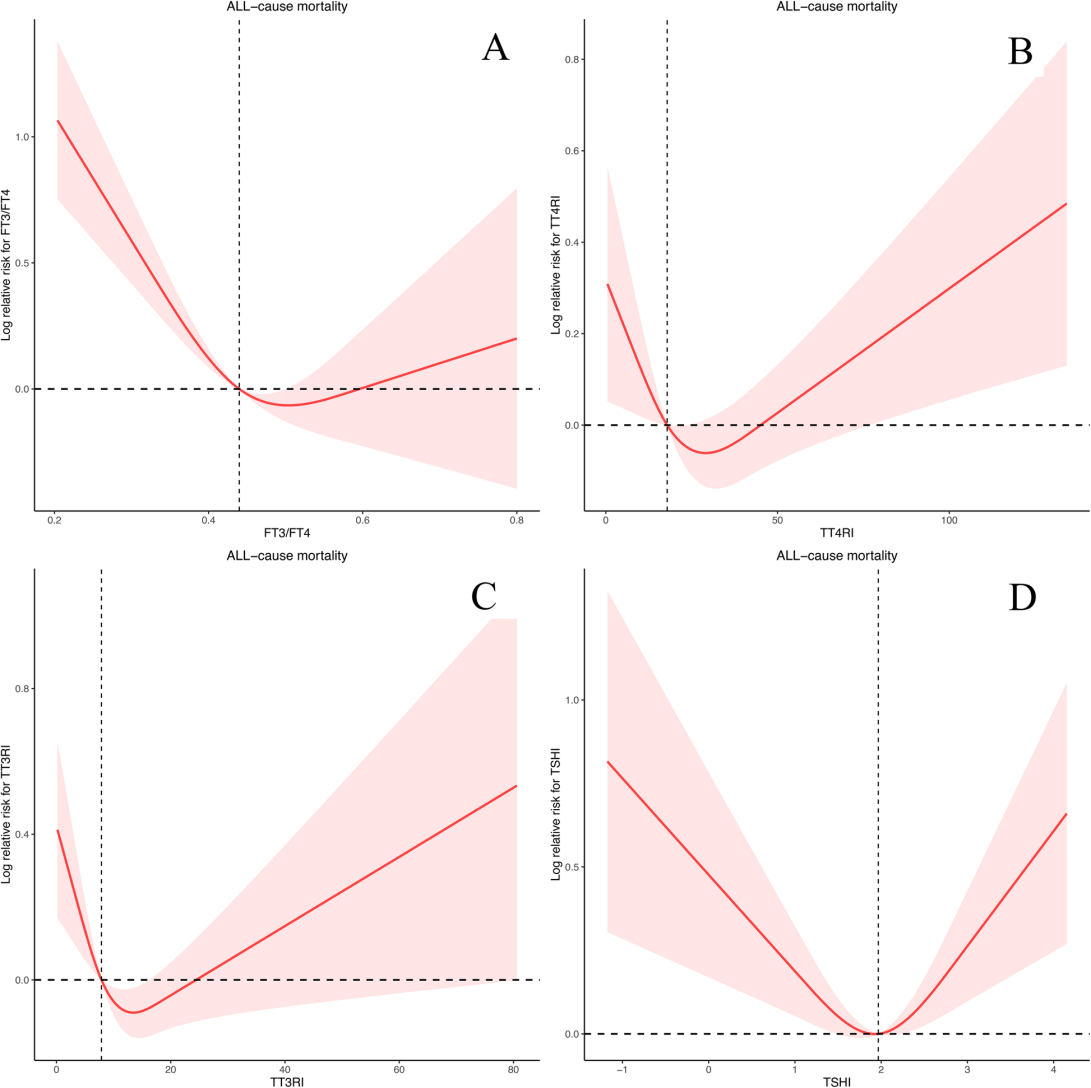
Association between FT3/FT4, TT4RI, TT3RI and TSHI with All-Cause Mortality among individuals with CKD using a Restricted Cubic Spline Regression Model

We designed 3 Cox regression models to investigate the independent role of FT3/FT4, TFQI_FT4_, TFQI_FT3_, TSHI, TT4RI and TT3RI in mortality. After multivariate adjustment including age, sex, race/ethnicity, education level, SBP, DBP, ALT, AST, BMI, history of hyperlipidemia or diabetes (Model 3), FT3/FT4 and TFQI_FT3_ were significantly associated with all-cause mortality. The multivariate-adjusted HR and 95% CI of FT3/FT4 and TFQI_FT3_ were 0.08(0.02,0.27) and 0.68(0.52,0.89). We further converted FT3/FT4, TFQI_FT4_, TFQI_FT3_, TSHI, TT4RI and TT3RI from a continuous variable to a categorical variable (quartiles) to conduct the sensitivity analysis. The HRs and 95% CIs from lowest to highest FT3/FT4 categories were 1.00 (reference), (HR = 0.62; 95% CI = 0.50–0.76, *P* < 0.0001), (HR = 0.54; 95% CI = 0.39–0.75, *P* < 0.001), and (HR = 0.63; 95% CI = 0.44–0.88, *P* = 0.01), respectively, for all-cause mortality (P trend = 0.003). The HRs and 95% CIs from lowest to highest TFQI_FT3_ categories were 1.00 (reference), (HR = 0.90; 95% CI = 0.67–1.22, *P* = 0.50), (HR = 0.70; 95% CI = 0.43–1.12, *P* = 0.14), and (HR = 0.72; 95% CI = 0.54–0.95, *P* = 0.02), respectively, for all-cause mortality (P trend = 0.008) (Table 4). However, we found no difference in cardiovascular mortality (Table 4). In crude Kaplan–Meier analyses, FT3/FT4, TFQI_FT4_, TFQI_FT3_, TT4RI, TT3RI and TSHI were also associated with all-cause mortality in CKD patients (Fig. 4A-F), but not associated with cardiovascular mortality (Supplementary Fig. 2). At the same time, we also conducted a sensitivity analysis, and after stratifying by age to analyze the K-M analysis, we found that the differences present in the KM analysis were more significant in the population aged 60 and above (Supplementary Fig. 3–5). There was a nonlinear association between FT3/FT4, TT4RI, TT3RI and TSHI with all-cause mortality (P for overall = 0, P for nonlinear = 0, Fig. 5A; P for overall = 0.0028, P for nonlinear = 0.005, Fig. 5B; P for overall = 0.0015, P for nonlinear = 0.0004, Fig. 5C; P for overall = 0.0003, P for nonlinear = 0.0001, Fig. 5D, respectively), but not TFQI_FT4_ and TFQI_FT3_ (P for nonlinear > 0.05). The changepoints of FT3/FT4, TT4RI, TT3RI and TSHI were 0.503, 29.354, 13.513, 1.930, respectively. There were nonlinear relationships between TFQI_FT3_ and TT4RI with cardiovascular mortality (P for overall = 0.0314, P for nonlinear = 0.0110, Supplementary Fig. 6 A; P for overall = 0.0298, P for nonlinear = 0.0087, Supplementary Fig. 6B), but not FT3/FT4, TFQI_FT4_, TT3RI and TSHI (P for overall > 0.05 or P for nonlinear > 0.05). We then examined the association between thyroid parameters within each risk group with all-cause and cardiovascular mortality in each stratum (Supplementary Table 12–13).

**Table 4:** Correlations of thyroid Homeostasis Parameters with the all-cause mortality and cardiovascular mortality among individuals with CKD

	**All-cause mortality**								**cardiovascular mortality**									
	Model 1		Model 2			Model 3				Model 1			Model 2			Model 3		
	HR (95% CI)	*P* value		HR (95% CI)	*P* value		HR (95% CI)	*P* value			HR (95% CI)	*P* value		HR (95% CI)	*P* value		HR (95% CI)	*P* value
FT3/FT4																		
Continuous	0.01(0.00,0.03)	< 0.0001		0.07(0.02,0.24)	< 0.0001		0.08(0.02,0.27)	< 0.0001			0.11(0.02,0.46)	0.003		0.17(0.03,0.96)	0.04		0.20(0.03,1.30)	0.09
Quartile 1	Ref	Ref		Ref	Ref		Ref	Ref			Ref	Ref		Ref	Ref		Ref	Ref
Quartile 2	0.53(0.42,0.66)	< 0.0001		0.60(0.48,0.75)	< 0.0001		0.62(0.50,0.76)	< 0.0001			0.60(0.36,0.98)	0.04		0.77(0.47,1.24)	0.28		0.79(0.47,1.32)	0.37
Quartile 3	0.35(0.24,0.51)	< 0.0001		0.52(0.37,0.73)	< 0.001		0.54(0.39,0.75)	< 0.001			0.86(0.52,1.44)	0.57		0.87(0.56,1.34)	0.52		0.90(0.59,1.36)	0.61
Quartile 4	0.33(0.23,0.48)	< 0.0001		0.62(0.44,0.88)	0.01		0.63(0.44,0.88)	0.01			0.45(0.29,0.72)	< 0.001		0.55(0.33,0.92)	0.02		0.57(0.32,1.02)	0.06
P for trend		< 0.0001			0.003			0.003				0.002			0.05			0.151
TFQI_FT4_																		
Continuous	2.42(1.77,3.31)	< 0.0001		1.48(1.04,2.10)	0.03		1.26(0.89,1.79)	0.19			1.27(0.66,2.44)	0.47		1.51(0.82,2.77)	0.19		1.22(0.60,2.46)	0.58
Quartile 1	Ref	Ref		Ref	Ref		Ref	Ref			Ref	Ref		Ref	Ref		Ref	Ref
Quartile 2	1.32(0.93,1.88)	0.12		0.99(0.69,1.42)	0.97		0.92(0.63,1.33)	0.65			1.25(0.77,2.01)	0.37		1.31(0.80,2.15)	0.28		1.46(0.94,2.28)	0.09
Quartile 3	1.50(1.00,2.23)	0.05		0.88(0.56,1.40)	0.60		0.77(0.49,1.19)	0.24			1.06(0.55,2.04)	0.86		1.46(0.91,2.33)	0.11		1.58(1.03,2.42)	0.04
Quartile 4	2.05(1.50,2.80)	< 0.0001		1.26(0.91,1.74)	0.16		1.10(0.79,1.52)	0.57			1.31(0.70,2.44)	0.40		1.71(0.99,2.95)	0.06		1.56(0.90,2.70)	0.11
P for trend		< 0.0001			0.257			0.676				0.598			0.093			0.282
TFQI_FT3_																		
Continuous	0.54(0.41,0.72)	< 0.0001		0.72(0.53,0.97)	0.03		0.68(0.52,0.89)	0.01			0.64(0.37,1.10)	0.11		0.88(0.51,1.54)	0.66		0.83(0.45,1.53)	0.55
Quartile 1	Ref	Ref		Ref	Ref		Ref	Ref			Ref	Ref		Ref	Ref		Ref	Ref
Quartile 2	0.78(0.59,1.02)	0.07		0.90(0.67,1.22)	0.50		0.90(0.67,1.22)	0.50			1.06(0.73,1.53)	0.76		1.10(0.75,1.62)	0.62		1.11(0.75,1.64)	0.60
Quartile 3	0.59(0.39,0.91)	0.02		0.70(0.43,1.12)	0.13		0.70(0.43,1.12)	0.14			1.19(0.72,1.96)	0.49		1.25(0.69,2.26)	0.46		1.02(0.46,2.28)	0.97
Quartile 4	0.57(0.43,0.75)	< 0.0001		0.78(0.57,1.06)	0.11		0.72(0.54,0.95)	0.02			0.56(0.34,0.92)	0.02		0.77(0.44,1.36)	0.37		0.71(0.38,1.33)	0.28
P for trend		< 0.0001			0.046			0.008				0.159			0.708			0.756
TT4RI																		
Continuous	1.01(1.01,1.01)	< 0.0001		1.00(1.00,1.01)	0.01		1.00(1.00,1.01)	0.08			1.00(0.99,1.01)	0.99		1.00(0.99,1.01)	0.55		1.00(0.98,1.01)	0.80
Quartile 1	Ref	Ref		Ref	Ref		Ref	Ref			Ref	Ref		Ref	Ref		Ref	Ref
Quartile 2	1.12(0.78,1.61)	0.53		0.91(0.63,1.31)	0.61		0.87(0.61,1.24)	0.44			1.31(0.91,1.91)	0.15		1.51(0.96,2.39)	0.08		1.54(0.97,2.46)	0.07
Quartile 3	1.05(0.74,1.49)	0.77		0.76(0.54,1.07)	0.11		0.67(0.48,0.94)	0.02			1.56(0.96,2.54)	0.07		1.66(0.93,2.99)	0.09		1.68(0.88,3.22)	0.12
Quartile 4	1.71(1.31,2.23)	< 0.0001		1.05(0.79,1.39)	0.74		0.96(0.73,1.25)	0.74			1.12(0.56,2.27)	0.74		1.54(0.82,2.86)	0.18		1.38(0.65,2.92)	0.40
P for trend		< 0.001			0.899			0.495				0.791			0.192			0.433
TT3RI																		
Continuous	1.01(1.00,1.01)	0.08		1.00(0.99,1.01)	0.59		1.00(0.99,1.01)	0.85			0.99(0.96,1.02)	0.60		1.00(0.98,1.03)	0.76		0.99(0.96,1.03)	0.76
Quartile 1	Ref	Ref		Ref	Ref		Ref	Ref			Ref	Ref		Ref	Ref		Ref	Ref
Quartile 2	0.76(0.55,1.04)	0.09		0.78(0.57,1.07)	0.12		0.81(0.59,1.12)	0.20			1.16(0.77,1.75)	1.16		1.36(0.83,2.23)	0.22		1.44(0.83,2.52)	0.20
Quartile 3	0.71(0.56,0.90)	0.005		0.63(0.48,0.81)	< 0.001		0.63(0.48,0.82)	< 0.001			1.20(0.86,1.67)	1.20		1.33(0.87,2.04)	0.19		1.22(0.70,2.15)	0.48
Quartile 4	1.09(0.84,1.40)	0.53		0.90(0.69,1.17)	0.41		0.84(0.67,1.07)	0.16			1.00(0.49,2.01)	1.00		1.40(0.72,2.72)	0.33		1.23(0.54,2.78)	0.62
P for trend		0.668			0.231			0.054				0.984			0.287			0.539
TSHI																		
Continuous	1.37(1.20,1.56)	< 0.0001		1.00(0.99,1.01)	0.23		1.01(0.90,1.14)	0.83			1.06(0.77,1.46)	0.71		1.20(0.90,1.60)	0.22		1.10(0.80,1.52)	0.55
Quartile 1	Ref	Ref		Ref	Ref		Ref	Ref			Ref	Ref		Ref	Ref		Ref	Ref
Quartile 2	0.91(0.65,1.28)	0.61		0.75(0.53,1.05)	0.10		0.71(0.50,1.01)	0.06			1.20(0.79,1.80)	0.39		1.22(0.74,2.01)	0.43		1.43(0.83,2.46)	0.20
Quartile 3	1.18(0.82,1.68)	0.37		0.80(0.57,1.12)	0.19		0.70(0.50,0.96)	0.03			1.47(0.95,2.28)	0.09		1.62(0.93,2.81)	0.09		1.71(0.92,3.18)	0.09
Quartile 4	1.65(1.25,2.17)	< 0.001		0.98(0.73,1.31)	0.88		0.89(0.67,1.18)	0.41			1.12(0.56,2.22)	0.75		1.50(0.80,2.82)	0.21		1.42(0.67,3.00)	0.36
P for trend		< 0.0001			0.780			0.596				0.738			0.181			0.343

Model 1: Non-adjusted

Model 2: Adjusted for age, sex, education level, race, SBP and DBP

Model 3: Adjusted for age, sex, education level, race, SBP, DBP, BMI, ALT, AST, urine iodine, DM, Hyperlipidemia, eGFR and UACR

## Discussion

Our study suggested that thyroid function and thyroid homeostasis parameters were associated with the prevalence of CKD and mortality among individuals with CKD.

Research suggested that Thyroid hormones (TH), especially triodothyronine (T3), can be considered as a marker for survival in patients with kidney disease [27].Our study also suggested that FT3 had a protective effect on all-cause mortality among individuals with CKD, which was consistent with the idea that FT3 had a protective effect on renal function [28, 29]. Combining the conclusions of this study with the results of previous studies, we hypothesized that the protective effect of FT3 on mortality first may be due to the protection of renal function and the prevention of further deterioration of renal function. On one hand, a retrospective study involving 309 patients showed that supplementation with levothyroxine in patients with subclinical hypothyroidism and chronic kidney disease (CKD) stages ≥ 2 can mitigate the decline in renal function [30]. On the other hand, in euthyroid rats treated with T3, there was an increase in the activity of the sodium–potassium adenosine triphosphate (ATP) pump (Na/K ATPase) as a response to elevated sodium reabsorption. [27, 31].

A Korean study reported that increased FT4 was associated with decreased eGFR [32]. In support of the Korean study was a Chinese study that observed that the risk of CKD was 1.763-fold higher in the highest quartiles of FT4 compared with the lowest quartile [33].On the basis of the conclusions of previous studies, our study also suggests that FT4 was a risk factor for the prevalence of CKD and all-cause mortality. This may be due to the abnormal elevation of FT4 exacerbates the decline of renal function in CKD patients. TSH showed the same trend as FT4. Previous studies have indicated that thyroid-stimulating hormone (TSH) is negatively correlated with renal function, possibly due to TSH-induced increases in urinary protein excretion [34–36]. Meanwhile, results from several large cross-sectional studies involving euthyroid participants have shown an inverse association between renal function and TSH level [37, 38]. In our research, we also considered TSH as a categorical variable. It is positive that we found a U-shaped curve of all-cause mortality in TSH and CKD patients. When the TSH is around 3.044 mIU/L, it will be the most beneficial point for CKD patients. However, our study confirmed that FT3, FT4 and TSH were not associated with cardiovascular mortality, which may also confirm that thyroid function may increase all-cause mortality by further reducing kidney function, rather than by increasing the risk of cardiovascular diseases.

Studied showed that CKD affected both hypothalamus–pituitary–thyroid axis and TH peripheral metabolism [39–42], which drives us to think about the influence of FT3/FT4 on the prevalence and prognosis of CKD. A prospective observational cohort study in a British population found that a low FT3/FT4 ratio was associated with frailty and long-term death in older hospitalised patients [43]. Many studies have also shown that FT3/FT4 is negatively correlated with disease mortality and adverse outcomes [44–46], which further suggests that we should pay extra attention to FT3/FT4 in some chronic diseases with poor prognosis. Recently, a study used FT3/FT4 as a continuous variable to explore the relationship between FT3/FT4 and 5-year mortality of patients with CKD, and proved that the FT3/FT4 ratio was significantly associated with a decreased 5-year all-cause mortality risk when FT3/FT4 ratio < 4.72 [47]. In our study, FT3/FT4 was also divided into quartiles to conduct a sensitivity analysis, which was consistent with most previous studies [1, 44, 48]. Meanwhile, our findings also demonstrated that FT3/FT4 was negatively associated with the prevalence of CKD. Which meant that FT3/FT4 could reflect the disease process of CKD patients to a certain extent.

In addition, we not only focused on the relationship between FT3/FT4 and all-cause mortality, but also explored the relationship between TFQIFT4, TFQIFT3, TT4RI, TT3RI, TSHI and mortality, which represented central sensitivity to thyroid hormones. Thyroid parameters offer a more comprehensive assessment of thyroid status compared to individual thyroid indicators. Chen et al. demonstrated elevated TSHI and TT4RI were associated with an increased prevalence of kidney disorders among type 2 diabetes patients [49]. Our study indicated that the relationship between these two parameters and all-cause mortality was U-shaped. Besides, TT4RI also had a non-linear relationship with cardiovascular mortality. Yang et al. substituting FT3 into the existing formulae and obtain new indices, TFQI_FT3_ and TT3RI, which also obtained strong correlations with renal function in euthyroid individuals. Similarly, our study also showed TFQI_FT3_, in the face of higher FT3, had a protective effect on the prevalence of CKD and all-cause mortality in patients with CKD, and had an inverted U-shaped relationship with cardiovascular mortality, which indicating that decreased peripheral thyroid sensitivity was significantly associated with reduced all-cause mortality. This provides a new avenue for further investigating FT3, TSHI and TT4RI. Meanwhile, thyroid dysfunction in CKD patients is characterized by low triiodothyronine (T3) levels with normal thyroxine (T4) and thyroid-stimulating hormone (TSH), forming a distinctive"euthyroid sick syndrome"pattern. This primarily results from impaired peripheral conversion of T4 to T3 due to uremic toxins, chronic inflammation, and malnutrition. The severity of these abnormalities typically correlates with CKD progression, becoming most pronounced in advanced stages (G4-G5).

A significant advantage of our study lies in the examination of the association between thyroid function and homeostasis parameters and all-cause mortality in individuals with CKD within a substantial, nationally representative sample of the US general population. Our study benefits from individualized linkage to the national mortality database, ensuring comprehensive documentation of death events through rigorous capture procedures. Nonetheless, our study is subject to several limitations that deserve consideration. Firstly, even though we have extensively accounted for confounding factors, their potential influence remains noteworthy, and future investigations should encompass a broader array of factors for adjustment. Secondly, as our study was conducted exclusively within the US population, replication across more diverse populations in the future would enhance its generalizability. Lastly, our study has not proved whether thyroid hormone supplementation can improve the prognosis and reduce the mortality of patients with CKD, and further studies are needed to confirm it.

## Conclusions

In conclusion, using a nationally representative database of US adults, this was the first population-based study to investigate the associations of thyroid function and homeostasis parameters and the prevalence of CKD and all-cause and cardiovascular mortality among CKD patients, which provided a substantial reference for the control of thyroid function and related parameters in CKD high-risk population and reduced mortality and improved survival in patients with CKD.

## Supplementary Information

Supplementary Material 1. Supplementary Figure 1 Kaplan-Meier survival estimates all-cause mortality across the quartiles of the thyroid hormones (FT3, FT4,TSH) among individuals with CKD in the age groups of 20-39 (A, B, C), 40-59 (D, E, F), and over 60 (G, H, I)

Supplementary Material 2. Supplementary Figure 2 Kaplan-Meier survival estimates cardiovascular mortality across the quartiles of the thyroid homeostasis parameters (FT3/FT4, TFQIFT4, TFQIFT3, TT4RI, TT3RI, TSHI) among individuals with CKD

Supplementary Material 3. Supplementary Figure 3 Kaplan-Meier survival estimates cardiovascular mortality across the quartiles of the thyroid homeostasis parameters (FT3/FT4, TFQIFT4, TFQIFT3, TT4RI, TT3RI, TSHI) among individuals with CKD in the age groups of 20-39

Supplementary Material 4. Supplementary Figure 4 Kaplan-Meier survival estimates cardiovascular mortality across the quartiles of the thyroid homeostasis parameters (FT3/FT4, TFQIFT4, TFQIFT3, TT4RI, TT3RI, TSHI) among individuals with CKD in the age groups of 40-59

Supplementary Material 5. Supplementary Figure 5 Kaplan-Meier survival estimates cardiovascular mortality across the quartiles of the thyroid homeostasis parameters (FT3/FT4, TFQIFT4, TFQIFT3, TT4RI, TT3RI, TSHI) among individuals with CKD in the age groups over 60

Supplementary Material 6. Supplementary Figure 6 Association between TFQIFT3 and TT4RI with cardiovascular mortality among individuals with CKD using a Restricted Cubic Spline Regression Model

Supplementary Material 7. 

Supplementary Material 8. 

Supplementary Material 9. 

Supplementary Material 10. 

Supplementary Material 11. 

Supplementary Material 12. 

Supplementary Material 13. 

Supplementary Material 14. 

Supplementary Material 15. 

Supplementary Material 16. 

Supplementary Material 17. 

Supplementary Material 18. 

Supplementary Material 19. 

## Data Availability

The data used in this study are from a public database at https://www.cdc.gov/nchs/nhanes/index.htm, which can be accessed by everyone through the links provided in the paper.

## Abbreviations

T4: Thyroxine
T3: Triodothyronine
TH: Thyroid hormones
FT3: Free triiodothyronine
FT4: Free thyroxine
HPT: Hypothalamus–pituitary–thyroid
TSH: Thyroid-stimulating hormone
TSHI: TSH index
TT4RI: Thyrotrophic T4 resistance index
TT3RI: Thyrotrophic T3 resistance index
TFQI_FT4_, TFQI_FT3_: Thyroid Feedback Quantile-based Index
FT3/FT4: FT3/FT4 ratio
T2D: Type 2 diabetes
CKD: Chronic kidney disease
KDIGO: Kidney Disease: Improving Global Outcomes
eGFR: Estimated glomerular filtration rate
UACR: Urinary albumin/creatinine ratio
NHANES: National Health and Nutrition Examination Survey
NCHS: National Center for Health Statistics
NDI: National Death Index
BMI: Body mass index
SBP: Systolic blood pressure
DBP: Diastolic blood pressure
ALT: Alanine aminotransferase
AST: Aspartate aminotransferase
DM: Diabetes mellitus
IGT: Impaired glucose tolerance
IFG: Impaired fasting glucose
TFQI: Thyroid Feedback Quantile-based Index
LDL-C: Low density lipoprotein
HDL-C: High density lipoprotein
ATP: Adenosine triphosphate
K-M: Kaplan–Meier
