# Supplementary figures and images for: Association between thyroid function and thyroid homeostasis parameters and the prevalence and all-cause and cardiovascular mortality of chronic kidney disease: a population-based study
Source: BMC Public Health. 2025 Aug 9;25:2715. doi: 10.1186/s12889-025-23695-z (PMC12335028; doi:10.1186/s12889-025-23695-z)

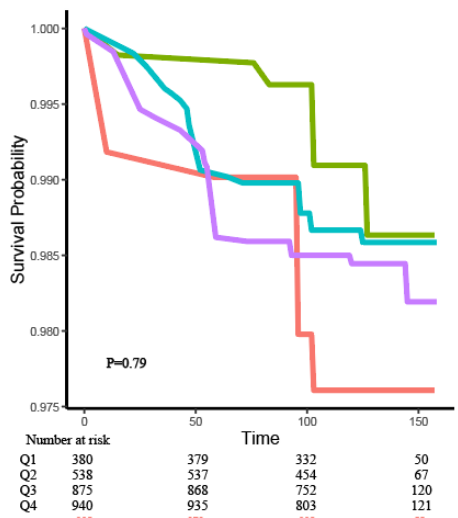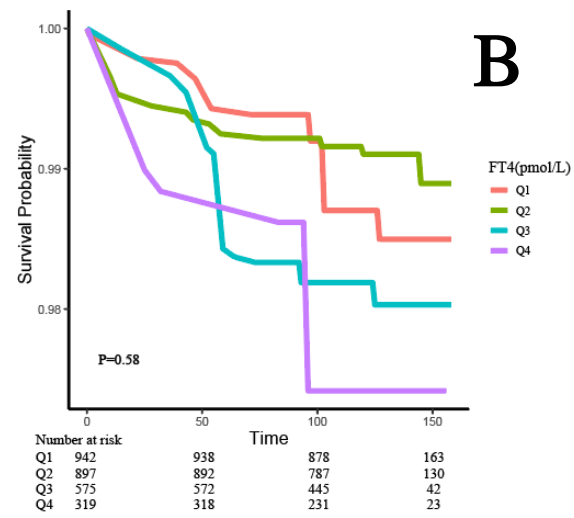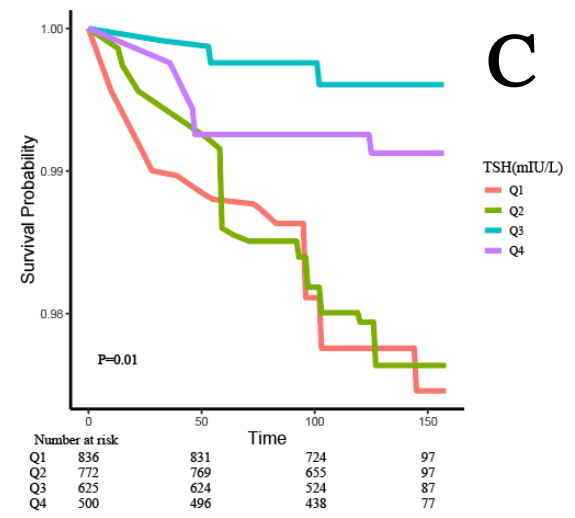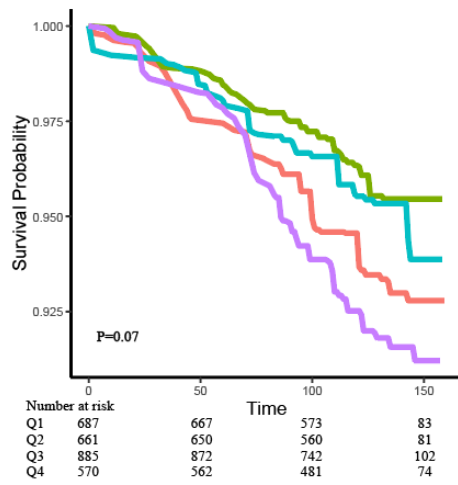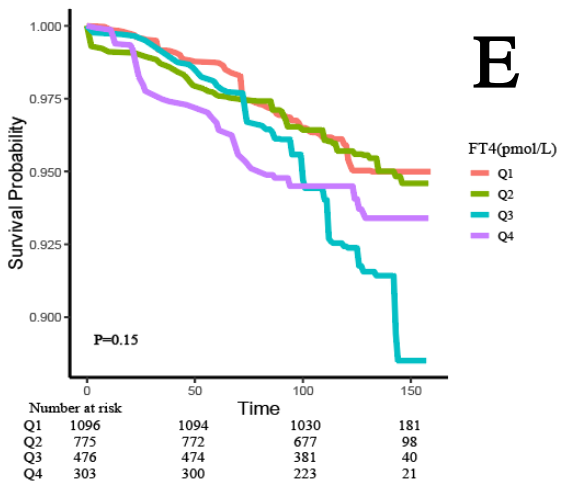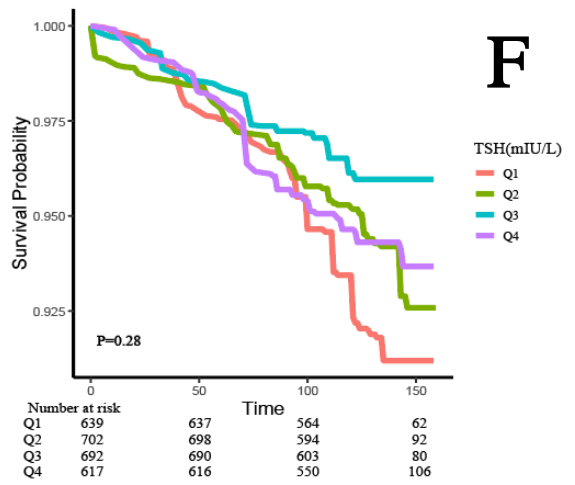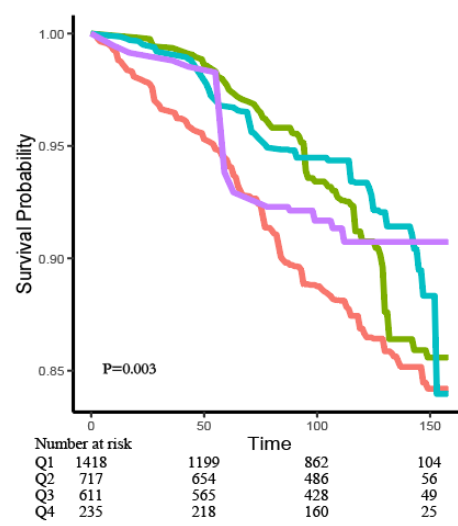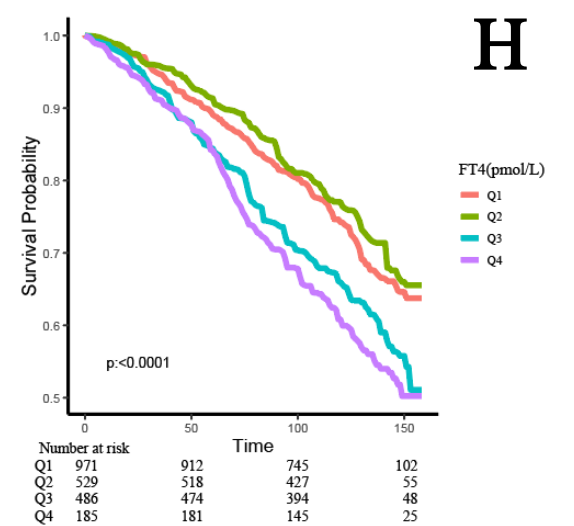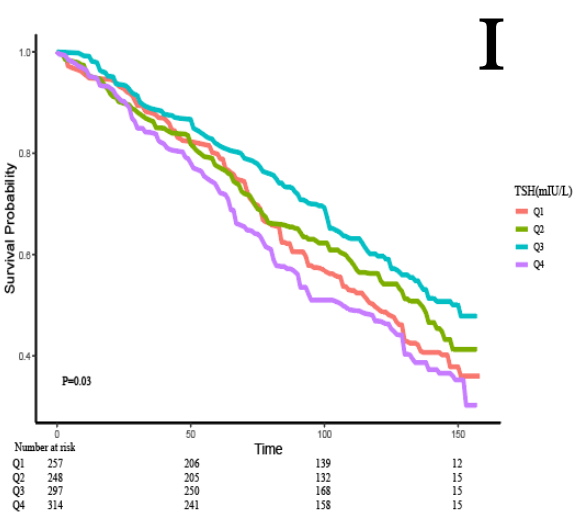

Supplement: Supplementary file 1 — Supplementary Material 1. Supplementary Figure 1 Kaplan-Meier survival estimates all-cause mortality across the quartiles of the thyroid hormones (FT3, FT4,TSH) among individuals with CKD in the age groups of 20-39 (A, B, C), 40-59 (D, E, F), and over 60 (G, H, I) [file 12889_2025_23695_MOESM1_ESM.pdf]

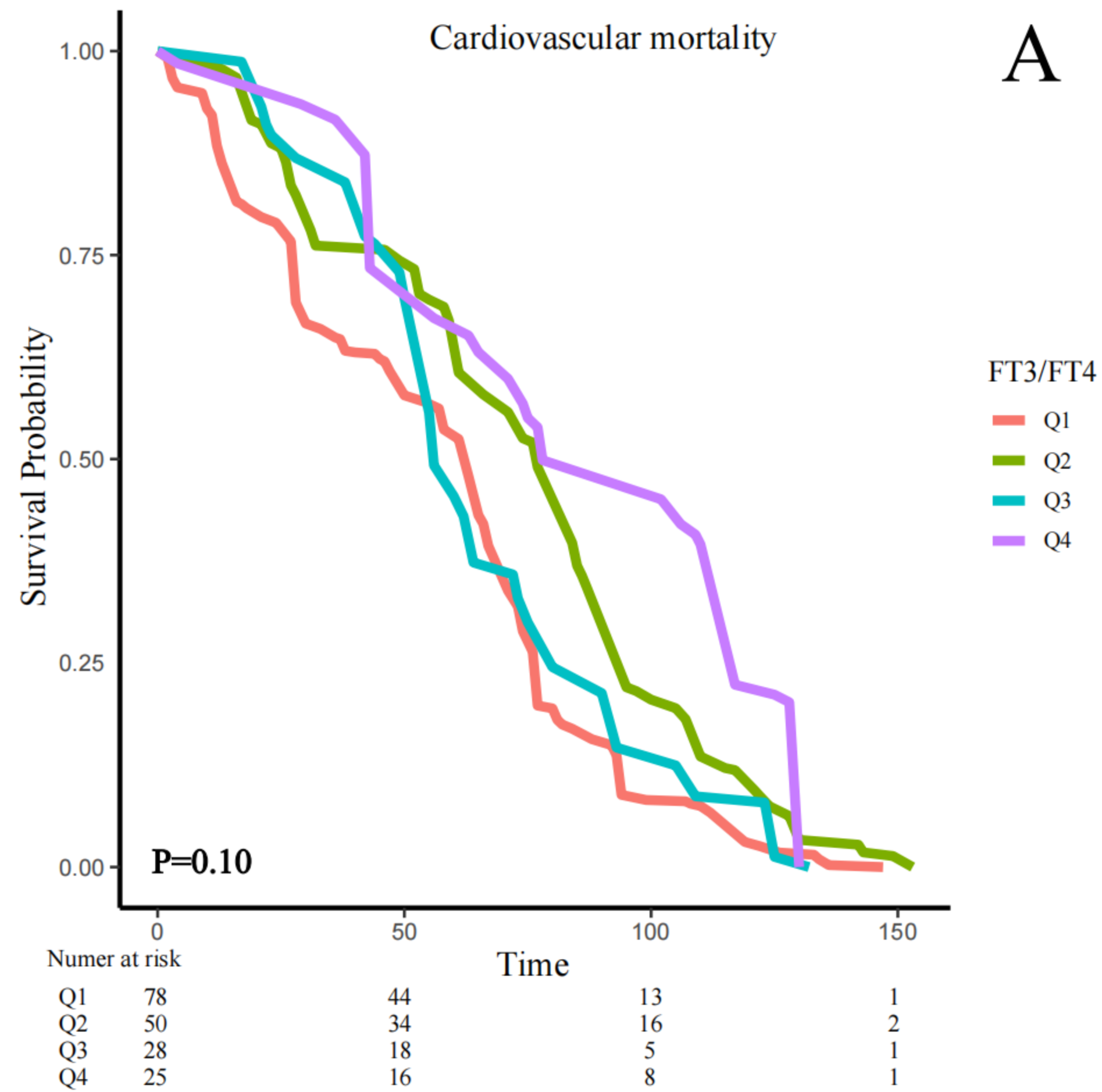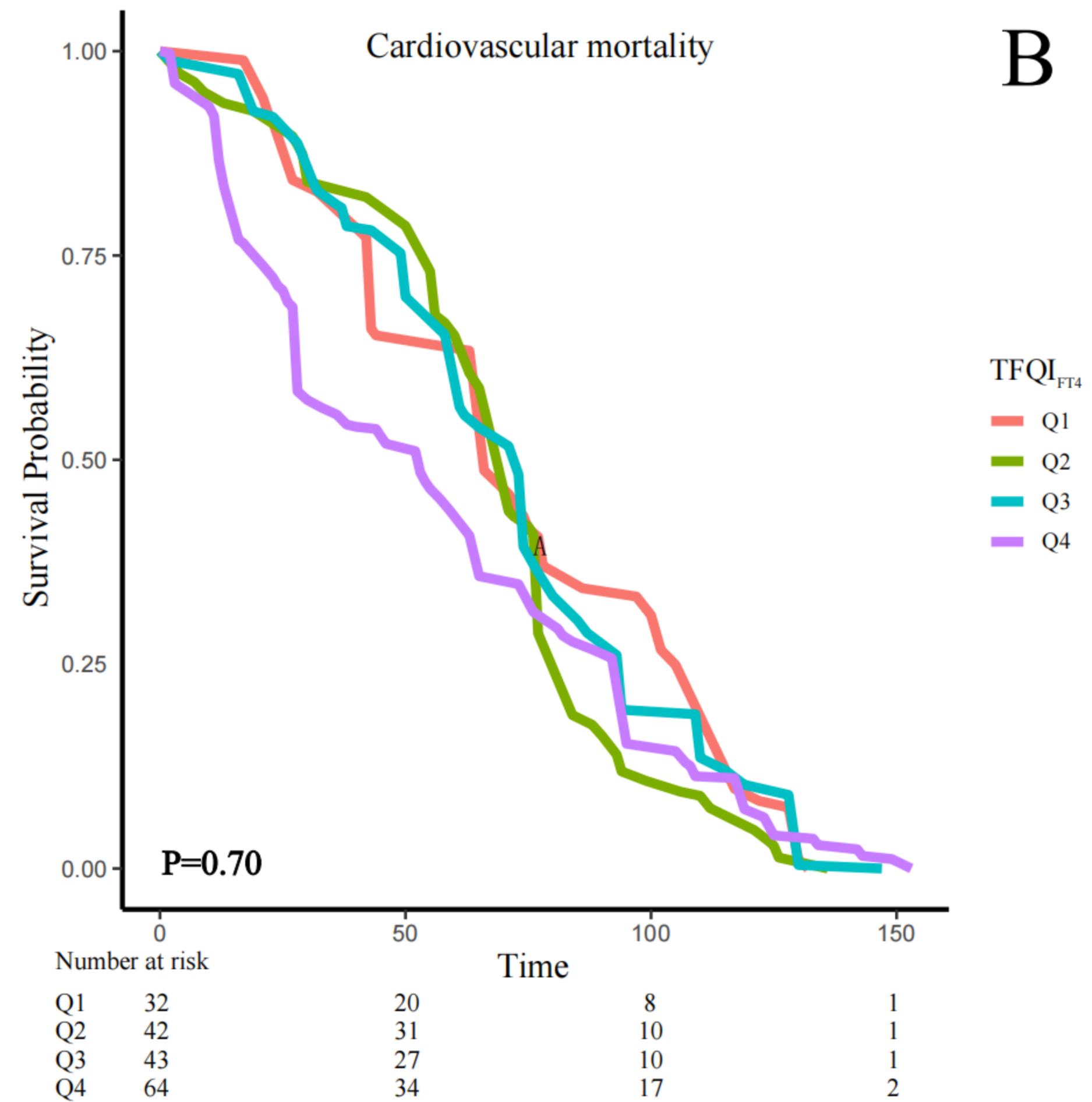

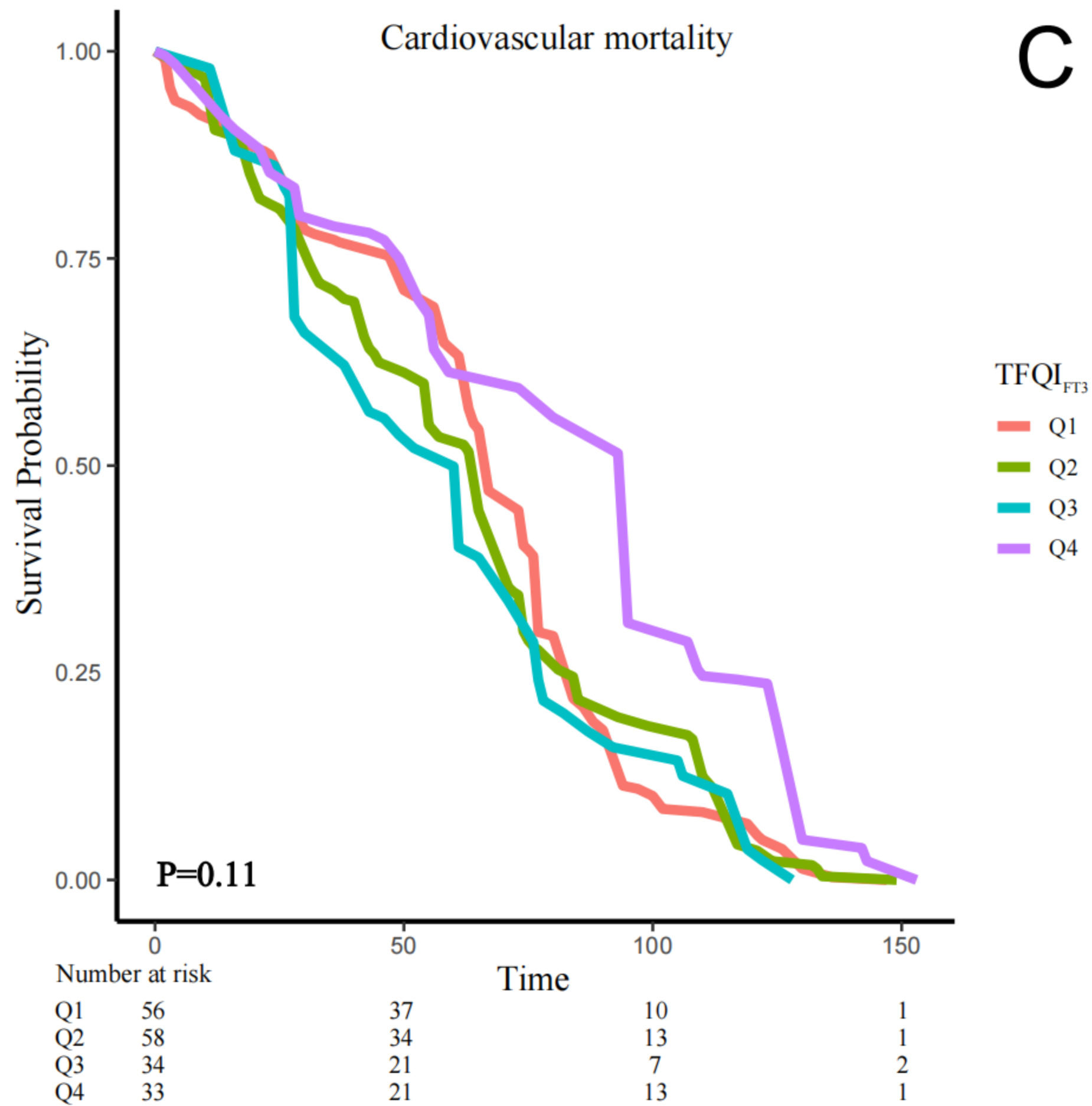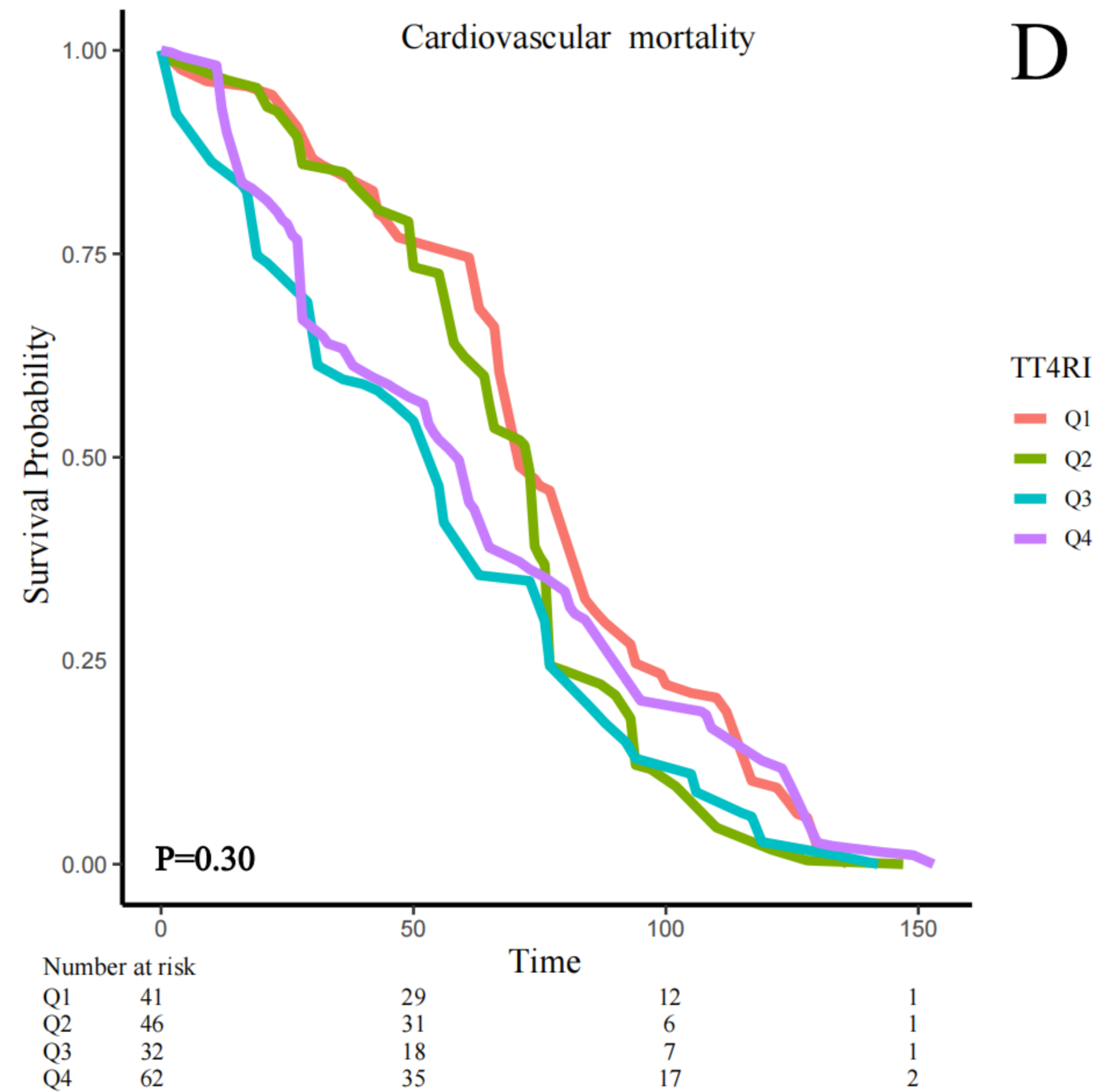

Cardiovascular mortality

E

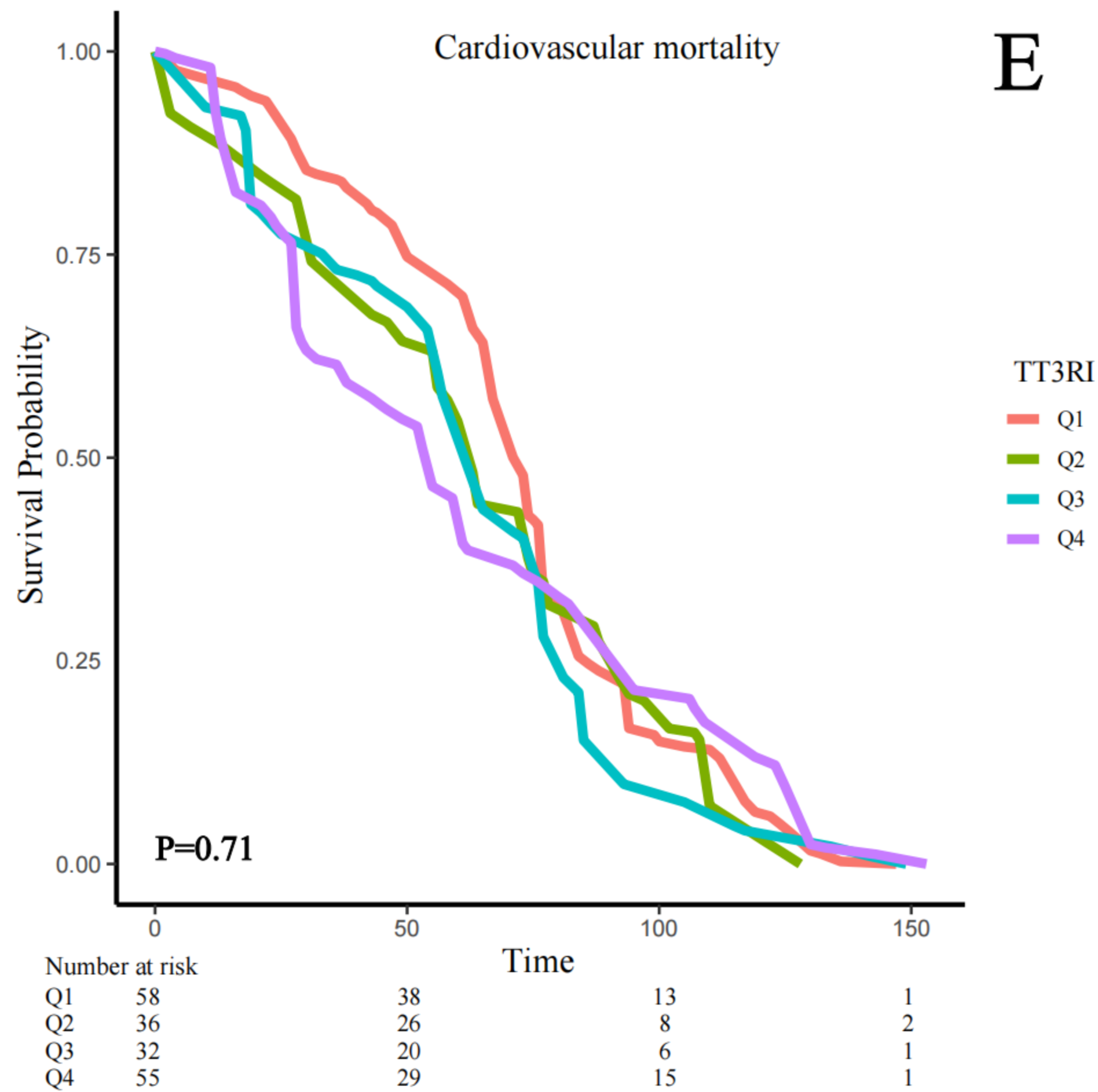

Cardiovascular mortality

F

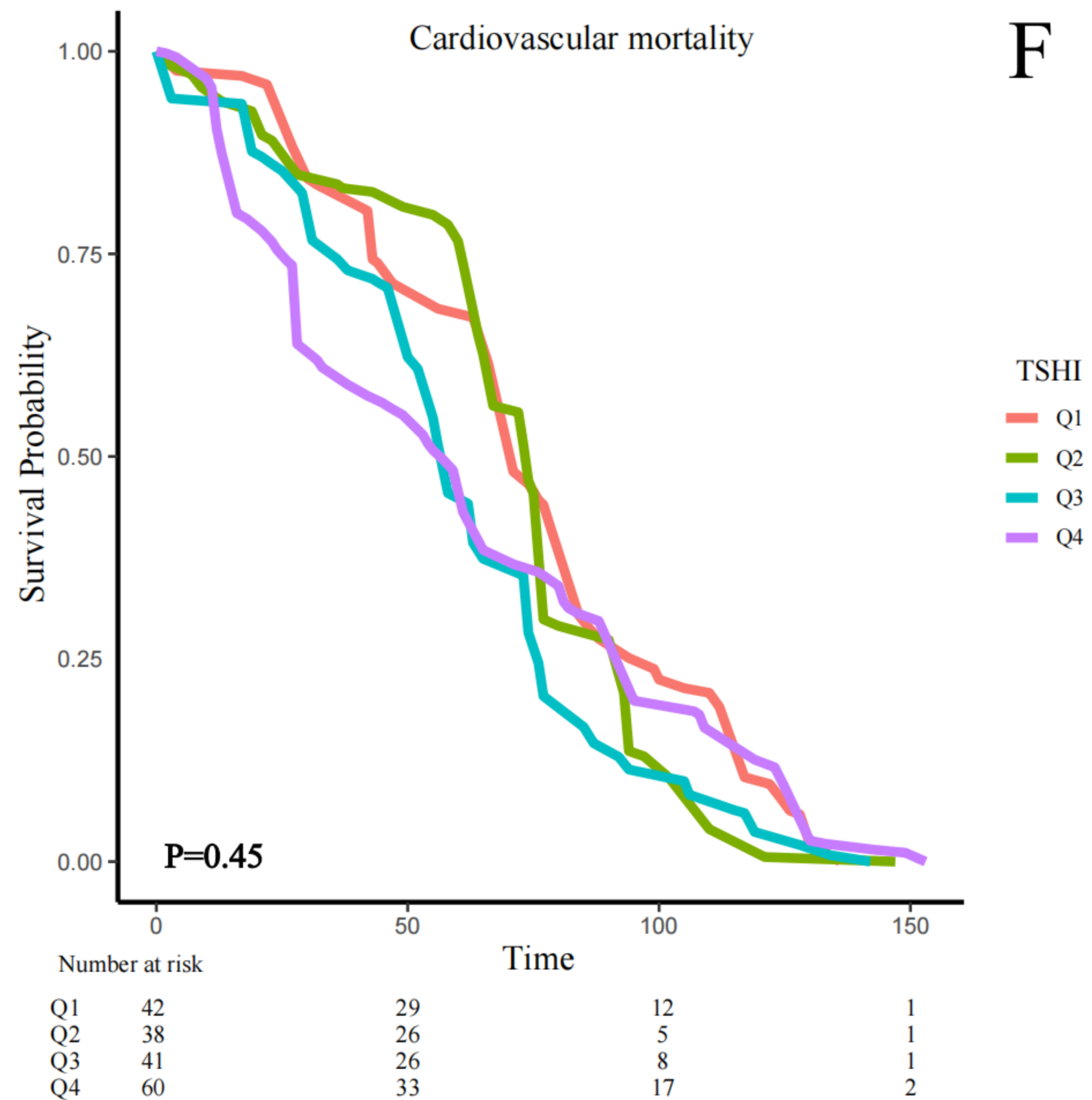

Supplement: Supplementary file 2 — Supplementary Material 2. Supplementary Figure 2 Kaplan-Meier survival estimates cardiovascular mortality across the quartiles of the thyroid homeostasis parameters (FT3/FT4, TFQIFT4, TFQIFT3, TT4RI, TT3RI, TSHI) among individuals with CKD [file 12889_2025_23695_MOESM2_ESM.pdf]

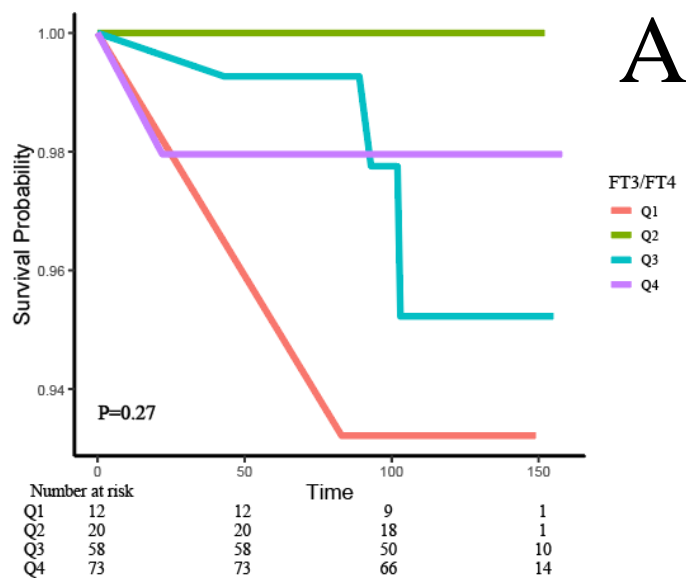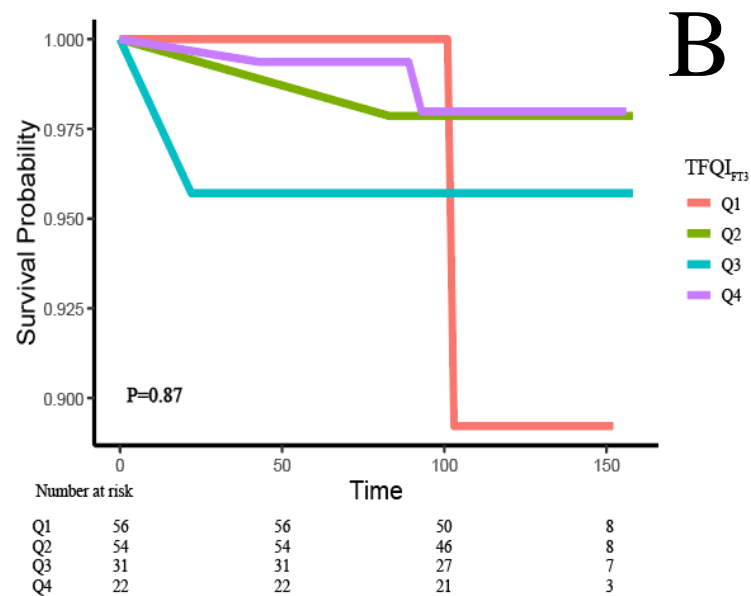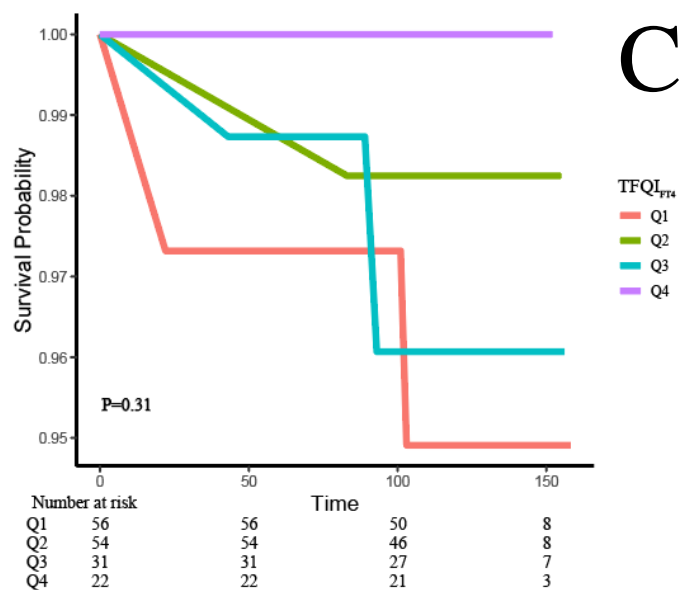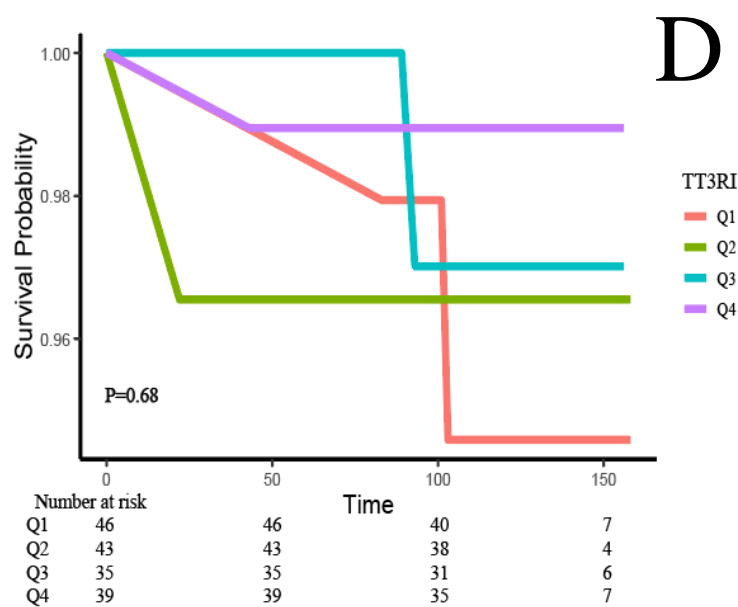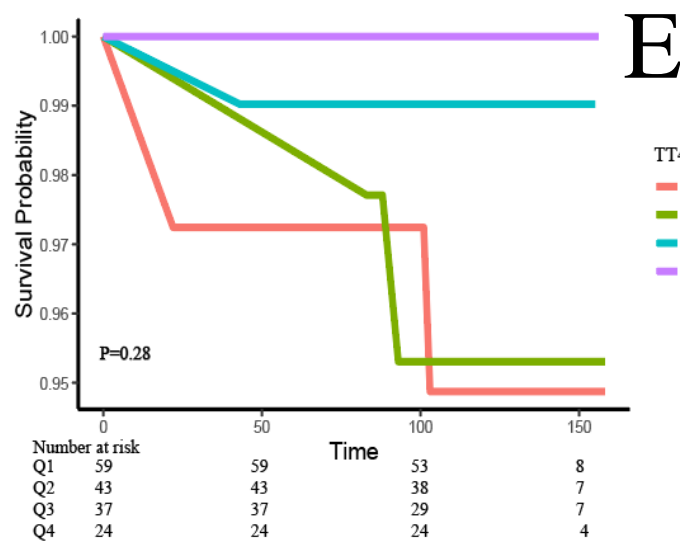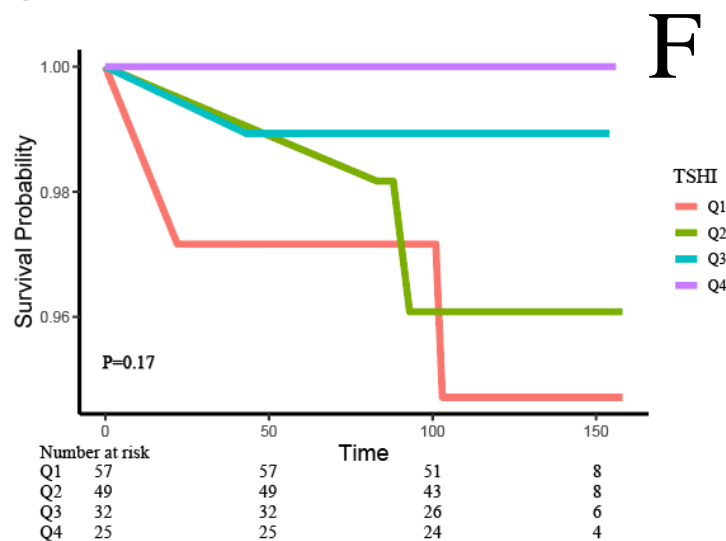

Supplement: Supplementary file 3 — Supplementary Material 3. Supplementary Figure 3 Kaplan-Meier survival estimates cardiovascular mortality across the quartiles of the thyroid homeostasis parameters (FT3/FT4, TFQIFT4, TFQIFT3, TT4RI, TT3RI, TSHI) among individuals with CKD in the age groups of 20-39 [file 12889_2025_23695_MOESM3_ESM.pdf]

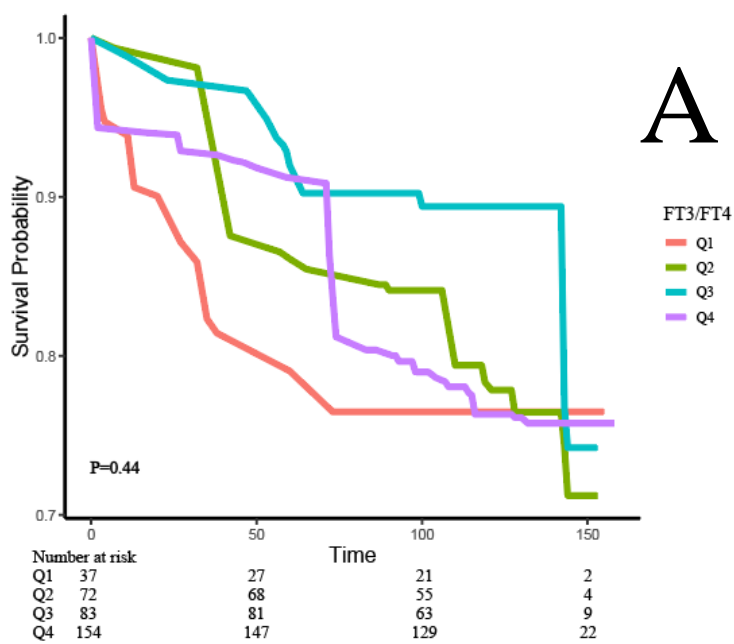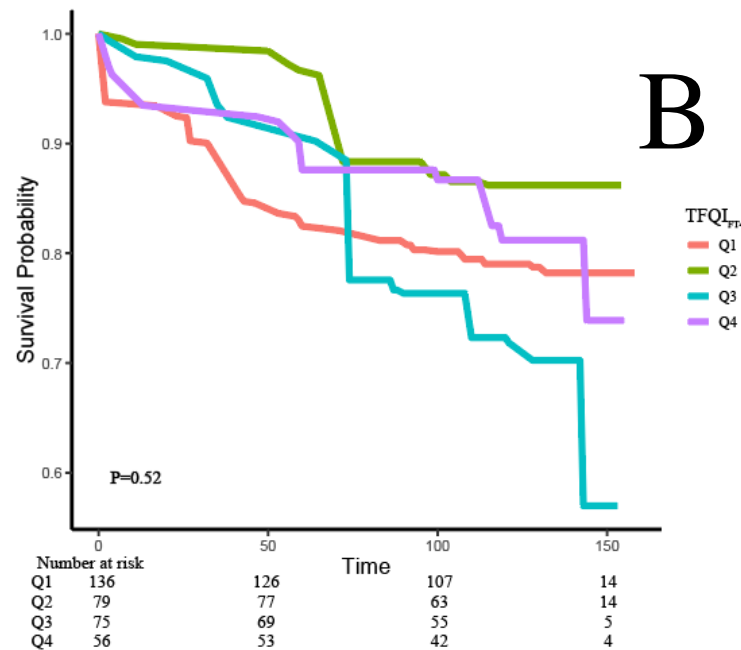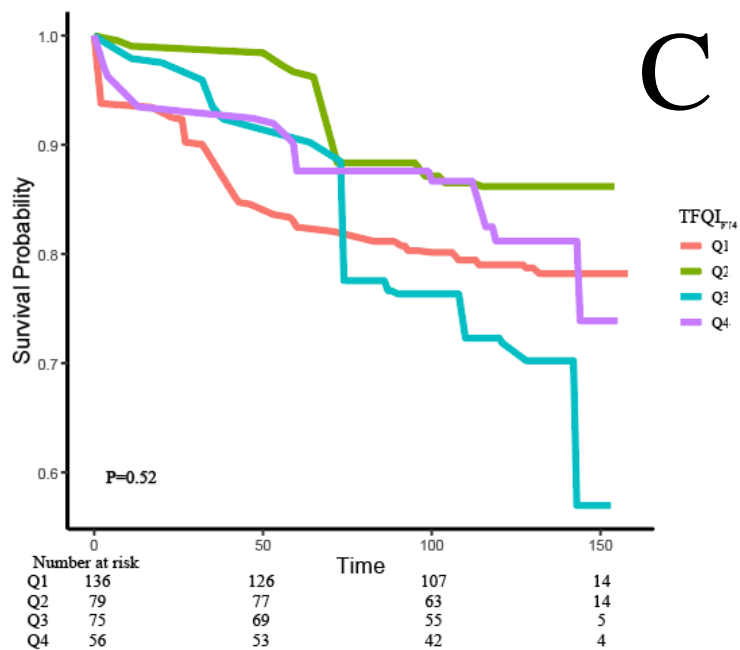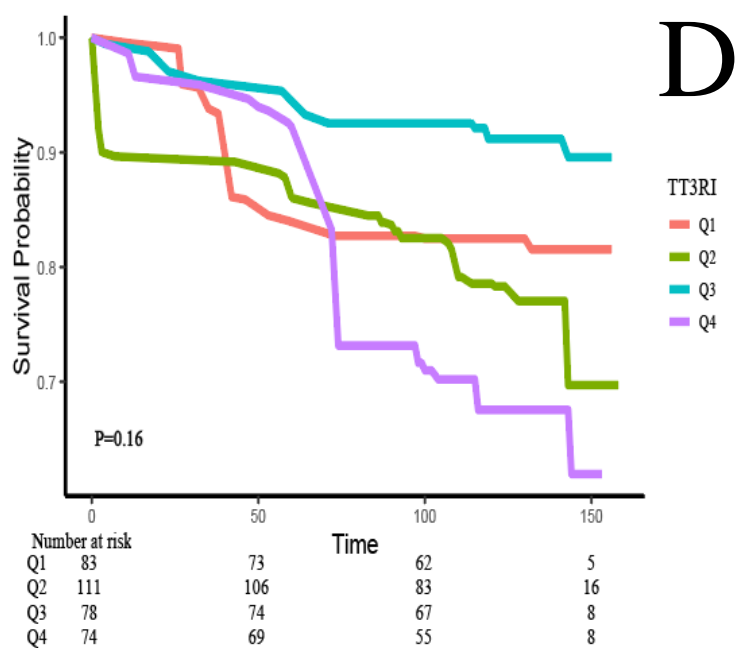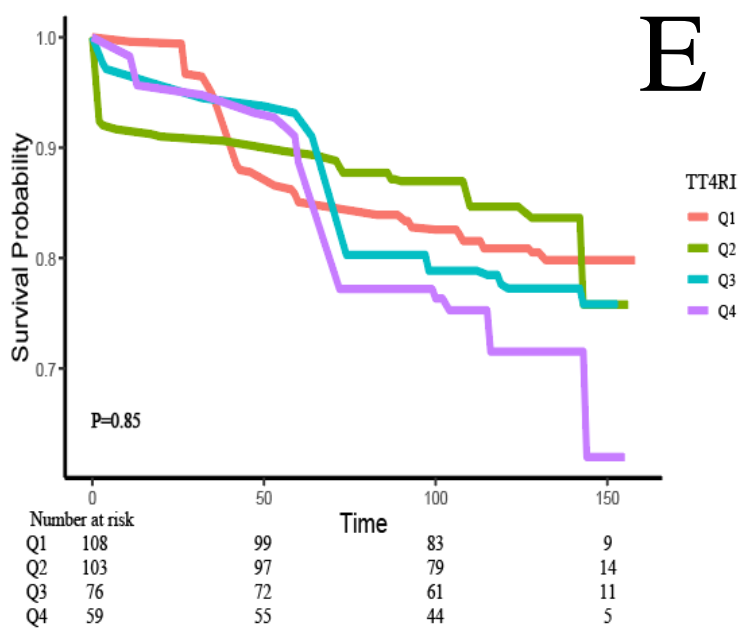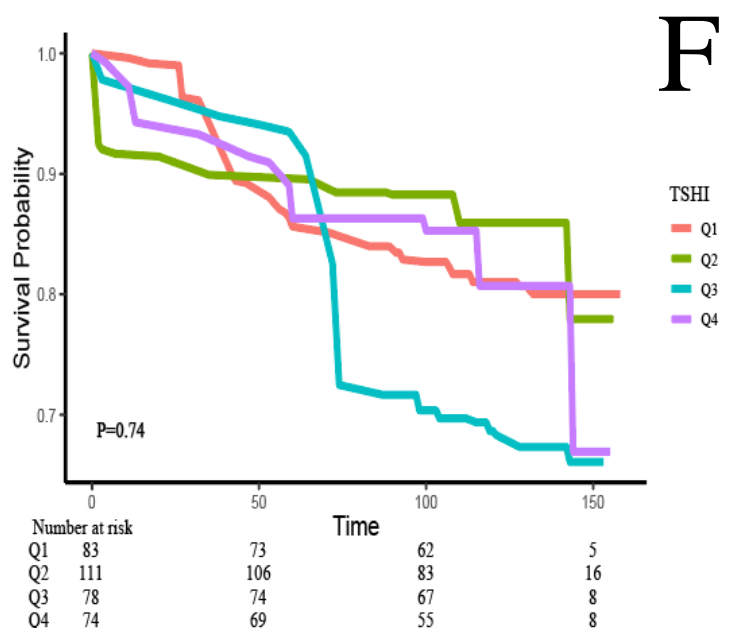

Supplement: Supplementary file 4 — Supplementary Material 4. Supplementary Figure 4 Kaplan-Meier survival estimates cardiovascular mortality across the quartiles of the thyroid homeostasis parameters (FT3/FT4, TFQIFT4, TFQIFT3, TT4RI, TT3RI, TSHI) among individuals with CKD in the age groups of 40-59 [file 12889_2025_23695_MOESM4_ESM.pdf]

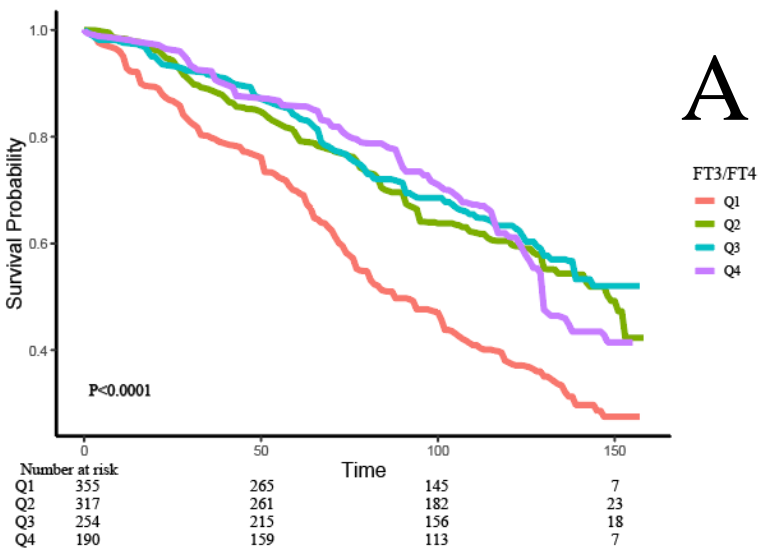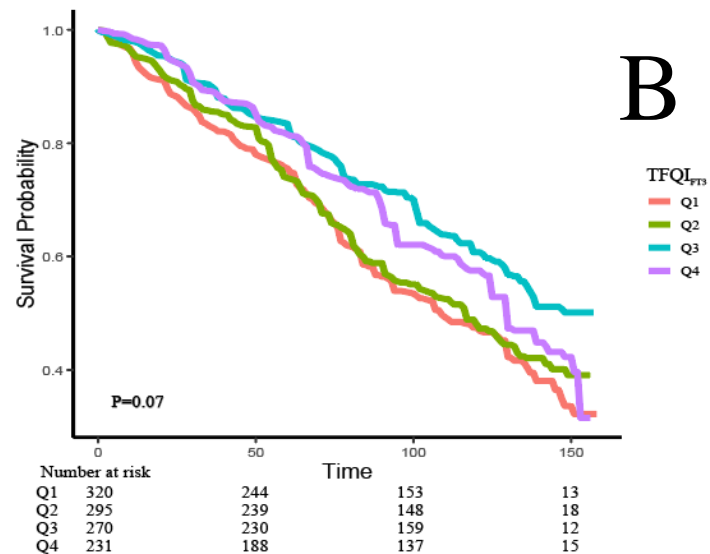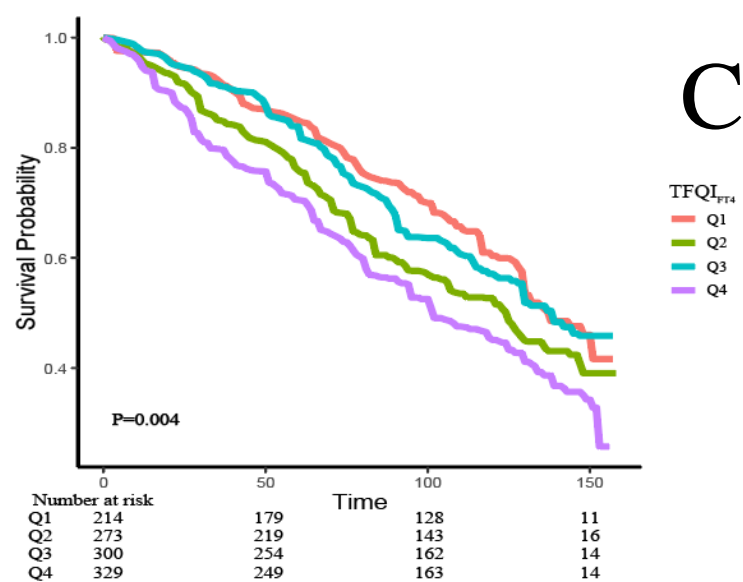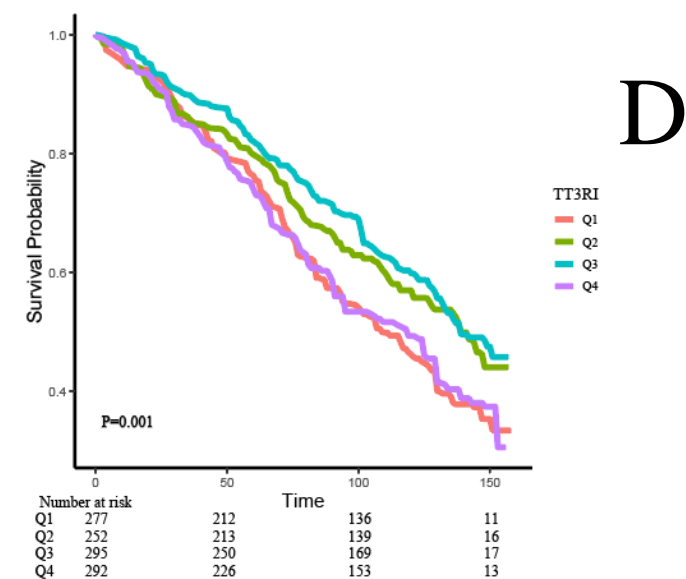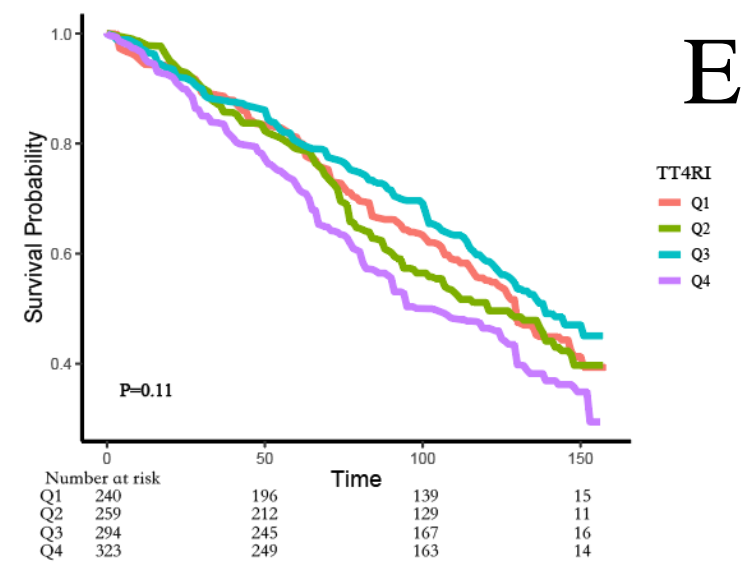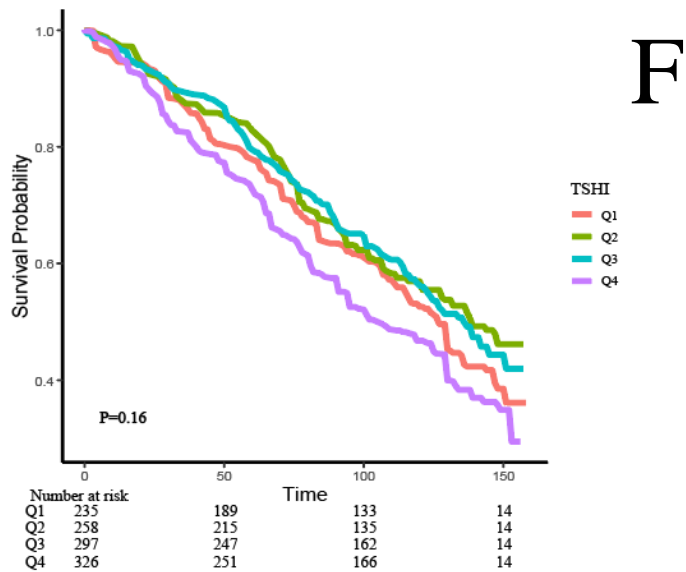

Supplement: Supplementary file 5 — Supplementary Material 5. Supplementary Figure 5 Kaplan-Meier survival estimates cardiovascular mortality across the quartiles of the thyroid homeostasis parameters (FT3/FT4, TFQIFT4, TFQIFT3, TT4RI, TT3RI, TSHI) among individuals with CKD in the age groups over 60 [file 12889_2025_23695_MOESM5_ESM.pdf]

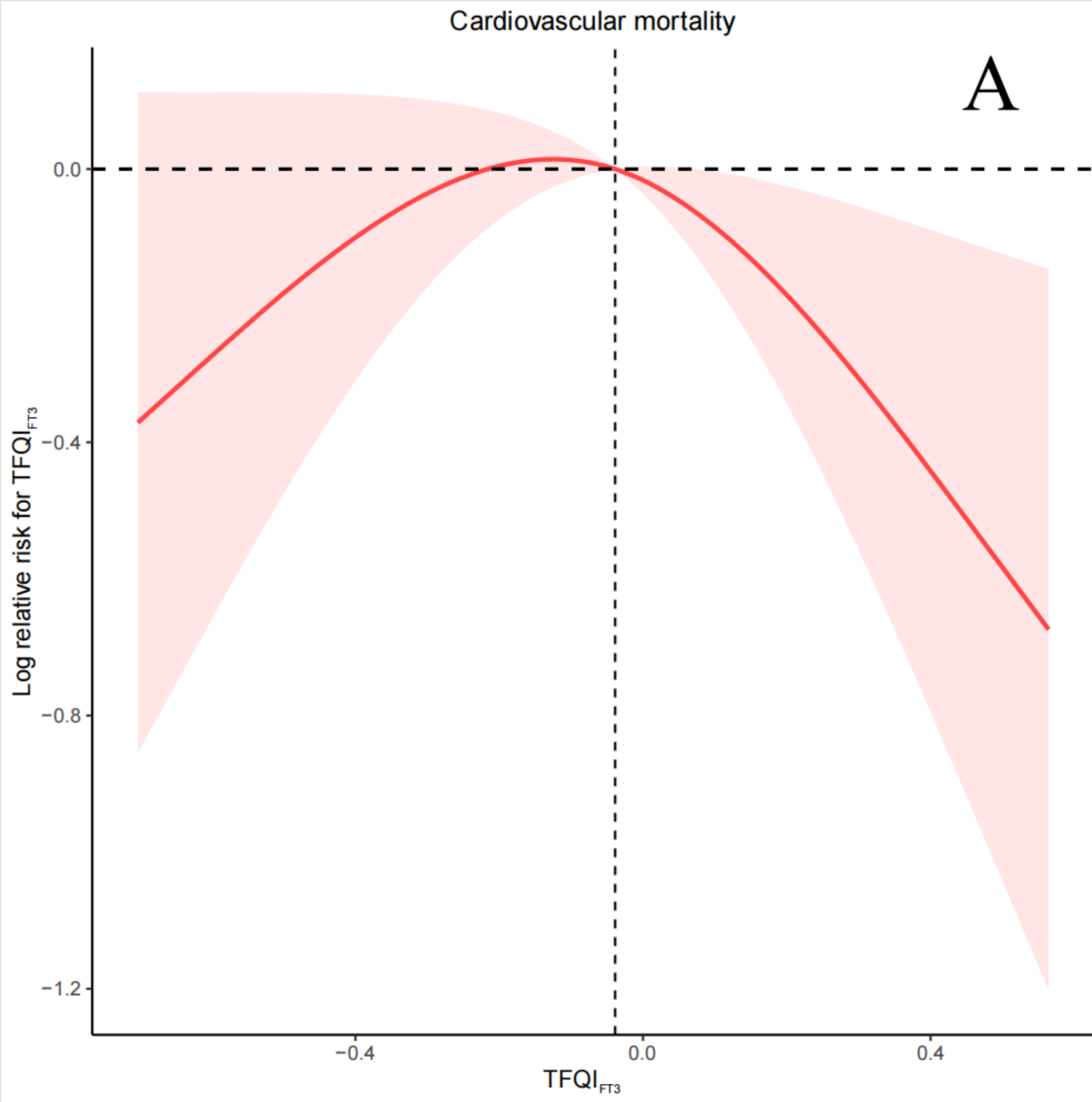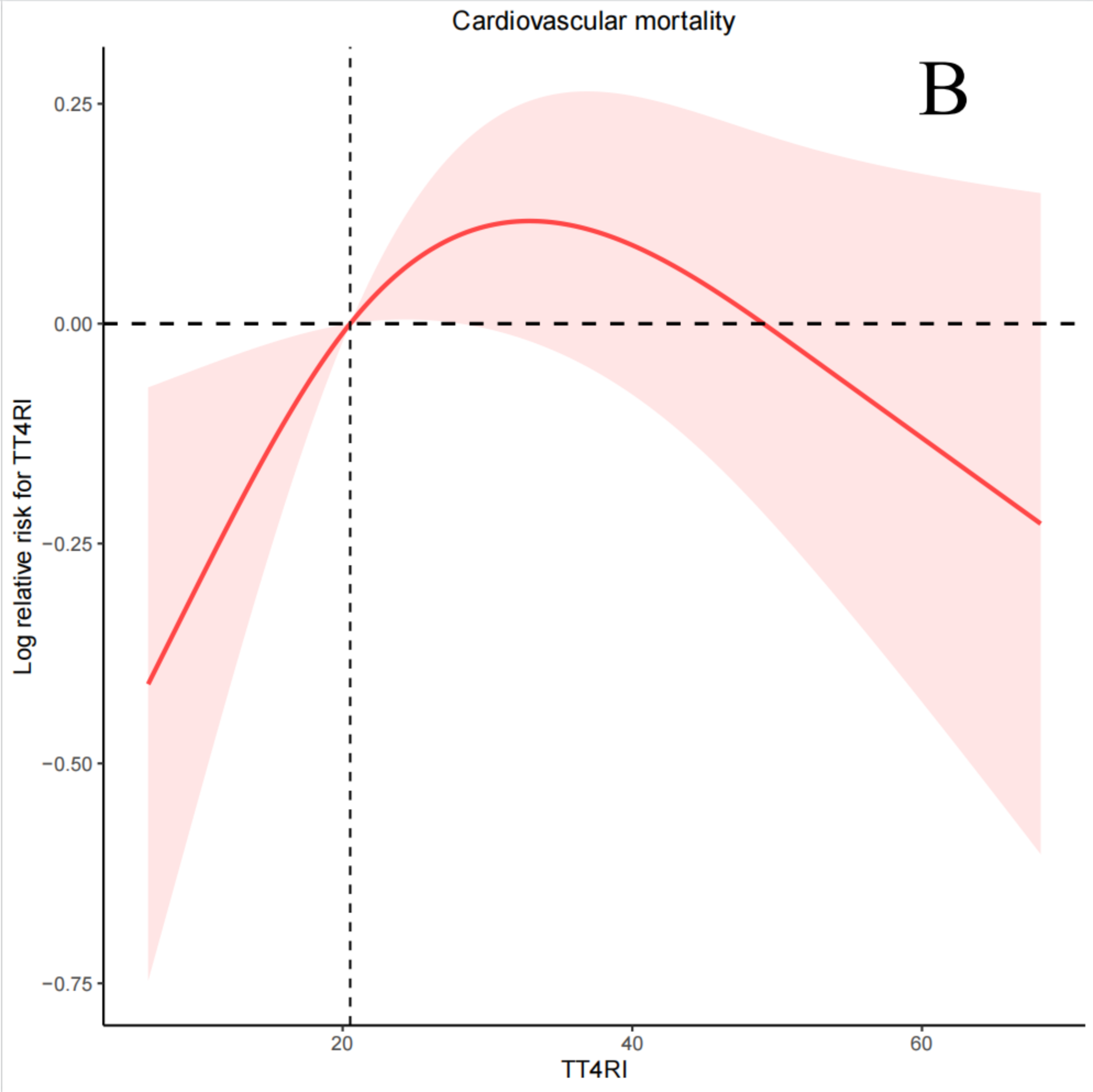

Supplement: Supplementary file 6 — Supplementary Material 6. Supplementary Figure 6 Association between TFQIFT3 and TT4RI with cardiovascular mortality among individuals with CKD using a Restricted Cubic Spline Regression Model [file 12889_2025_23695_MOESM6_ESM.pdf]
