# Supplementary material for: Association between thyroid function and thyroid homeostasis parameters and the prevalence and all-cause and cardiovascular mortality of chronic kidney disease: a population-based study
Source: BMC Public Health. 2025 Aug 9;25:2715. doi: 10.1186/s12889-025-23695-z (PMC12335028; doi:10.1186/s12889-025-23695-z)
Supplement: Supplementary file 7 — Supplementary Material 7. [file 12889_2025_23695_MOESM7_ESM.docx]

**Supplementary Table 1 Characteristics and thyroid parameters of the U.S. population represented in the NHANES sample (N = 1,625)**

|  | % | FT4 | P | FT3 | P | TSH | P | FT3/FT4 | P | TFQI_FT4_ | P | TFQI_FT3_ | P | TSHI | P | TT4RI | P | TT3RI | P |
| --- | --- | --- | --- | --- | --- | --- | --- | --- | --- | --- | --- | --- | --- | --- | --- | --- | --- | --- | --- |
| All |  | 11.03 |  | 4.64 |  | 2.17 |  | 0.44 |  | 0.06 |  | 0.02 |  | 1.96 |  | 22.61 |  | 9.81 |  |
| Age group |  |  | < 0.0001* |  | < 0.0001* |  | < 0.001* |  | < 0.0001* |  | < 0.0001* |  | < 0.0001* |  | < 0.0001* |  | < 0.0001* |  | 0.06 |
| 20-40 | 10.03 | 10.28 |  | 5.15 |  | 1.81 |  | 0.51 |  | -0.08 |  | 0.19 |  | 1.76 |  | 18.09 |  | 9.16 |  |
| 40-60 | 21.29 | 10.35 |  | 4.86 |  | 1.85 |  | 0.49 |  | -0.07 |  | 0.08 |  | 1.76 |  | 18.11 |  | 8.78 |  |
| >60 | 68.68 | 11.46 |  | 4.44 |  | 2.38 |  | 0.41 |  | 0.13 |  | -0.04 |  | 2.08 |  | 25.41 |  | 10.36 |  |
| Sex |  |  | 0.05* |  | < 0.001* |  | 0.83 |  | < 0.001* |  | 0.66 |  | < 0.0001* |  | 0.89 |  | 0.59 |  | 0.53 |
| female | 52.49 | 11.18 |  | 4.56 |  | 2.18 |  | 0.43 |  | 0.06 |  | -0.03 |  | 1.96 |  | 22.87 |  | 9.69 |  |
| male | 47.51 | 10.82 |  | 4.75 |  | 2.16 |  | 0.46 |  | 0.05 |  | 0.10 |  | 1.96 |  | 22.26 |  | 9.96 |  |
| Race |  |  | 0.01* |  | 0.003* |  | < 0.0001* |  | < 0.0001* |  | < 0.001* |  | < 0.001* |  | < 0.0001* |  | < 0.0001* |  | < 0.0001* |
| Mexican american | 13.48 | 10.51 |  | 4.89 |  | 2.00 |  | 0.48 |  | -0.02 |  | 0.11 |  | 1.84 |  | 20.27 |  | 9.48 |  |
| Non-hispanic black | 22.40 | 10.61 |  | 4.61 |  | 1.88 |  | 0.45 |  | -0.05 |  | -0.07 |  | 1.77 |  | 17.78 |  | 8.30 |  |
| Non-hispanic white | 49.91 | 11.12 |  | 4.59 |  | 2.30 |  | 0.44 |  | 0.09 |  | 0.03 |  | 2.03 |  | 24.31 |  | 10.36 |  |
| Other hispanic | 8.98 | 10.88 |  | 5.02 |  | 2.05 |  | 0.47 |  | -0.01 |  | 0.09 |  | 1.90 |  | 20.86 |  | 9.64 |  |
| Other race | 5.23 | 11.77 |  | 4.65 |  | 1.48 |  | 0.41 |  | 0.02 |  | -0.10 |  | 1.76 |  | 16.92 |  | 6.80 |  |

FT3 triiodothyronine (pg/mL), FT4 free thyroxine (pmol/L), TSH thyroid-stimulating hormone (mIU/L), TSHI TSH index, TT4RI thyrotrophic T4 resistance index, TT3RI thyrotrophic T3 resistance index, TFQIFT4, TFQIFT3 thyroid Feedback Quantile-based Index, FT3/FT4 FT3/FT4 ratio

P values are for differences between groups and were calculated from linear regression models.

*p<0.05
