# Supplementary material for: Association between thyroid function and thyroid homeostasis parameters and the prevalence and all-cause and cardiovascular mortality of chronic kidney disease: a population-based study
Source: BMC Public Health. 2025 Aug 9;25:2715. doi: 10.1186/s12889-025-23695-z (PMC12335028; doi:10.1186/s12889-025-23695-z)
Supplement: Supplementary file 9 — Supplementary Material 9. [file 12889_2025_23695_MOESM9_ESM.docx]

**Supplementary Table 3 Characteristics of subjects by quartiles of serum FT4 levels.**

| Variable | Quartile 1 | Quartile 2 | Quartile 3 | Quartile 4 | P value |
| --- | --- | --- | --- | --- | --- |
| Age (years) | 57.75(1.16) | 61.04(1.13) | 61.03(1.55) | 68.07(0.93) | **< 0.0001*** |
| Age-group (years) |  |  |  |  | **< 0.0001*** |
| >=60 | 280(47.31) | 308(60.00) | 248(62.29) | 280(77.24) |  |
| 18-39 | 58(18.13) | 49(16.36) | 40(15.85) | 16( 5.63) |  |
| 40-59 | 141(34.56) | 91(23.63) | 68(21.86) | 46(17.12) |  |
| Sex |  |  |  |  | 0.27 |
| female | 243(54.84) | 226(54.68) | 187(55.15) | 197(64.07) |  |
| male | 236(45.16) | 222(45.32) | 169(44.85) | 145(35.93) |  |
| Race |  |  |  |  | 0.07 |
| mexican american | 67(9.03) | 67(7.85) | 46(5.85) | 39(5.39) |  |
| non-hispanic black | 101(10.75) | 117(18.55) | 82(13.43) | 64(11.40) |  |
| non-hispanic white | 246(71.48) | 207(64.84) | 175(70.54) | 183(71.20) |  |
| other hispanic | 52(5.83) | 35(3.86) | 31(5.26) | 28(4.85) |  |
| other race - including multi-racial | 13(2.92) | 22(4.89) | 22(4.93) | 28(7.15) |  |
| Education levels |  |  |  |  | 0.21 |
| College graduate or above | 176(45.46) | 178(49.65) | 148(51.33) | 127(39.48) |  |
| High school or equivalent | 209(42.82) | 170(35.95) | 140(35.82) | 149(45.77) |  |
| Under high school | 93(11.72) | 98(14.41) | 68(12.86) | 64(14.75) |  |
| Tg (ng/mL) | 19.19(1.70) | 16.71(1.50) | 18.70(1.45) | 19.70(2.81) | 0.74 |
| TgAb (IU/mL) | 20.46(7.89) | 13.64(4.72) | 13.65(7.61) | 17.23(7.05) | 0.87 |
| TPOAb (IU/mL) | 31.91(9.36) | 18.37(4.43) | 22.19(5.51) | 18.68(5.75) | 0.61 |
| BMI (kg/m^2^) | 30.72(0.63) | 29.45(0.51) | 30.49(0.57) | 29.59(0.55) | 0.27 |
| ALT (U/L) | 26.04(0.89) | 25.15(1.51) | 26.62(2.02) | 20.68(0.54) | **< 0.0001*** |
| AST (U/L) | 27.79(0.81) | 27.20(1.03) | 31.41(4.08) | 25.02(0.42) | **0.003*** |
| SBP (mmHg) | 129.89(1.31) | 130.10(1.54) | 134.32(1.42) | 129.67(1.57) | 0.09 |
| DBP (mmHg) | 71.17(1.02) | 68.36(1.03) | 71.27(1.12) | 64.14(1.26) | **< 0.0001*** |
| Urine iodine (ug/L) | 78.04(1.94) | 73.14(1.95) | 71.97(2.02) | 63.90(2.38) | **< 0.001*** |
| Urine iodine-group |  |  |  |  | 0.54 |
| ≤100 | 131(28.71) | 135(28.09) | 105(30.10) | 98(31.75) |  |
| 100-199 | 144(31.58) | 142(33.14) | 123(33.82) | 93(24.94) |  |
| ＞199 | 202(39.71) | 171(38.78) | 127(36.08) | 150(43.31) |  |
| eGFR (mL/min/1.73m^2^) | 78.04(1.94) | 73.14(1.95) | 71.97(2.02) | 63.90(2.38) | **< 0.001*** |
| UACR (mg/g) | 153.03(24.34) | 174.68(24.24) | 234.27(49.24) | 194.26(31.93) | 0.43 |
| Hyperlipidemia |  |  |  |  | 0.28 |
| No | 68(14.50) | 82(20.39) | 59(14.86) | 53(14.96) |  |
| Yes | 411(85.50) | 366(79.61) | 297(85.14) | 289(85.04) |  |
| DM |  |  |  |  | 0.04* |
| No | 244(57.10) | 222(55.65) | 180(62.52) | 151(48.34) |  |
| IGT | 22(5.08) | 18(4.41) | 18(3.43) | 13(3.38) |  |
| IFG | 22(7.01) | 26(7.00) | 16(3.14) | 14(2.65) |  |
| Yes | 188(30.82) | 181(32.94) | 142(30.90) | 164(45.63) |  |

Data were presented as mean±SD or median (interquartile ranges) for continuous variables, and numbers (proportions) for categorical variables.

Tg thyroglobulin, TgAb thyroglobulin antibody, TPOAb thyroid peroxidase antibody, BMI body mass index, ALT glutamic-pyruvic transaminase, AST glutamic oxaloacetic transaminase, SBP systolic pressure, DBP diastolic pressure, eGFR estimated glomerular filtration rate, UACR urinary albumin to creatinine ratio, DM diabetes mellitus, IGT impaired glucose tolerance, IFG impaired fasting glucose.

*p<0.05
