# Supplementary material for: Association between thyroid function and thyroid homeostasis parameters and the prevalence and all-cause and cardiovascular mortality of chronic kidney disease: a population-based study
Source: BMC Public Health. 2025 Aug 9;25:2715. doi: 10.1186/s12889-025-23695-z (PMC12335028; doi:10.1186/s12889-025-23695-z)
Supplement: Supplementary file 17 — Supplementary Material 17. [file 12889_2025_23695_MOESM17_ESM.docx]

**Supplementary Table 11 Baseline characteristics of included participants in NHANES 2007-2012 across different CKD progression risk strata based on KDIGO classification..**

| **Variable** | **Low risk** | **Moderate risk** | **High risk** | **Very high risk** | **P** **value** |  |
| --- | --- | --- | --- | --- | --- | --- |
| **Age (years)** | 45.45±0.36 | 59.24±0.88 | 66.69±1.15 | 70.51±1.43 | < 0.0001 |  |
| **Age-group (years)** |  |  |  |  | < 0.0001 |  |
| **>=60** | 1868(19.50) | 653(55.08) | 254(74.71) | 181(80.50) |  |  |
| **18-39** | 2574(38.41) | 135(17.35) | 21( 7.65) | 6( 4.23) |  |  |
| **40-59** | 2459(42.09) | 275(27.57) | 47(17.64) | 20(15.26) |  |  |
| **Sex** |  |  |  |  | 0.001 |  |
| **female** | 3468(50.57) | 569(57.78) | 154(52.81) | 109(59.79) |  |  |
| **male** | 3433(49.43) | 494(42.22) | 168(47.19) | 98(40.21) |  |  |
| **Race** |  |  |  |  | 0.004 |  |
| **mexican american** | 1162( 8.25) | 148( 6.58) | 50(10.12) | 19( 5.85) |  |  |
| **non-hispanic black** | 1301( 9.96) | 234(13.39) | 71(12.56) | 55(16.07) |  |  |
| **non-hispanic white** | 3131(68.58) | 524(70.37) | 158(66.16) | 104(67.21) |  |  |
| **other hispanic** | 793(5.90) | 102(5.03) | 22(3.76) | 21(6.87) |  |  |
| **other race** | 514(7.31) | 55(4.63) | 21(7.40) | 8(3.99) |  |  |
| **Education levels** |  |  |  |  | < 0.0001 |  |
| **College graduate or above** | 3442(61.02) | 448(51.50) | 104(35.31) | 66(31.31) |  |  |
| **High school or equivalent** | 2708(33.59) | 423(37.12) | 141(48.03) | 86(45.82) |  |  |
| **Under high school** | 748( 5.40) | 192(11.38) | 75(16.66) | 53(22.87) |  |  |
| **Tg (ng/mL)** | 15.44±0.56 | 17.01±1.02 | 23.34±3.20 | 22.70±2.57 | 0.01 |  |
| **TgAb (IU/mL)** | 8.71±1.28 | 14.08±3.86 | 24.94±13.01 | 17.99±8.12 | 0.001 |  |
| **TPOAb (IU/mL)** | 22.29± 1.75 | 24.98±4.07 | 18.02±4.53 | 17.25±5.85 | 0.8 |  |
| **BMI (kg/m^2^)** | 28.45±0.13 | 30.03±0.36 | 29.68±0.53 | 30.10±0.88 | < 0.0001 |  |
| **ALT (U/L)** | 26.25±0.39 | 25.43±0.81 | 23.80±1.36 | 22.00±3.01 | < 0.0001 |  |
| **AST (U/L)** | 26.17±0.25 | 28.43±1.49 | 27.01±1.32 | 25.74±2.02 | 0.36 |  |
| **SBP (mmHg**) | 119.17±0.38 | 129.87±0.88 | 133.10±1.66 | 135.75±2.60 | < 0.0001 |  |
| **DBP (mmHg**) | 70.81±0.34 | 69.44±0.61 | 69.94±0.98 | 62.78±1.82 | < 0.0001 |  |
| **eGFR(mL/min/1.73m²)** | 98.35±0.50 | 80.83±1.22 | 63.04±1.85 | 32.21±0.98 | < 0.0001 |  |
| **uACR (mg/g)** | 7.66±0.13 | 54.96±3.14 | 400.74±57.25 | 637.53±134.38 | < 0.0001 |  |
| **Urine iodine (ug/L)** | 292.49±67.12 | 380.82±54.84 | 258.39±30.57 | 528.42±150.97 | < 0.0001 |  |
| **Urine iodine-group** |  |  |  |  | < 0.0001 |  |
| **≤100** | 2204(34.59) | 305(29.32) | 84(27.05) | 53(36.46) |  |  |
| **100-199** | 2209(32.05) | 319(31.58) | 92(27.75) | 66(32.34) |  |  |
| **＞199** | 2459(42.09) | 275(27.57) | 47(17.64) | 20(15.26) |  |  |
| **Hyperlipidemia** |  |  |  |  | < 0.0001 |  |
| **No** | 1892(28.01) | 177(17.03) | 46(14.06) | 36(14.52) |  |  |
| **Yes** | 5009(71.99) | 886(82.97) | 276(85.94) | 171(85.48) |  |  |
| **DM** |  |  |  |  | < 0.0001 |  |
| **No** | 5200(80.66) | 569(60.60) | 124(43.54) | 88(44.70) |  |  |
| **IGT** | 319(4.53) | 54(4.84) | 11(2.49) | 5(1.91) |  |  |
| **IFG** | 313(3.87) | 53(5.18) | 14(3.82) | 10(5.61) |  |  |
| **Yes** | 989(10.93) | 384(29.37) | 172(50.14) | 104(47.78) |  |  |
| **FT3 (pg/mL)** | 3.19±0.01 | 3.08±0.02 | 2.93±0.04 | 2.74±0.03 | < 0.0001 |  |
| **FT4 (pmol/L)** | 10.30±0.07 | 10.88±0.12 | 11.11±0.28 | 11.79±0.31 | < 0.0001 |  |
| **TSH (mIU/L)** | 2.05±0.07 | 2.04±0.07 | 2.35±0.13 | 2.67±0.19 | 0.01 |  |
| **FT3/FT4** | 0.49±0.00 | 0.46±0.00 | 0.43±0.01 | 0.38±0.01 | < 0.0001 |  |
| **TFQI_FT4_** | 0.05±0.01 | 0.11±0.02 | 0.19±0.03 | 0.33±0.04 | < 0.0001 |  |
| **TFQI_FT3_** | 0.04±0.01 | -0.03±0.02 | -0.09±0.03 | -0.17±0.03 | < 0.0001 |  |
| **TT4RI** | 19.93±0.47 | 20.90±0.68 | 24.22±1.30 | 30.32±2.54 | < 0.0001 |  |
| **TT3RI** | 9.83±0.33 | 9.50±0.31 | 10.32±0.53 | 10.92±0.64 | < 0.001 |  |
| **TSHI** | 1.81±0.02 | 1.88±0.04 | 2.05±0.05 | 2.25±0.08 | 0.31 | |

Data were presented as mean±SD or median (interquartile ranges) for continuous variables, and numbers (proportions) for categorical variables.

Tg thyroglobulin, TgAb thyroglobulin antibody, TPOAb thyroid peroxidase antibody, BMI body mass index, ALT glutamic-pyruvic transaminase, AST glutamic oxaloacetic transaminase, SBP systolic pressure, DBP diastolic pressure, eGFR estimated glomerular filtration rate, UACR urinary albumin to creatinine ratio, DM diabetes mellitus, IGT impaired glucose tolerance, IFG impaired fasting glucose, FT3 triiodothyronine, FT4 free thyroxine, TSH thyroid-stimulating hormone, TSHI TSH index, TT4RI thyrotrophic T4 resistance index, TT3RI thyrotrophic T3 resistance index, TFQI_FT4_, TFQI_FT3_ thyroid Feedback Quantile-based Index, FT3/FT4 FT3/FT4 ratio

*p<0.05
