# Supplementary material for: Association between thyroid function and thyroid homeostasis parameters and the prevalence and all-cause and cardiovascular mortality of chronic kidney disease: a population-based study
Source: BMC Public Health. 2025 Aug 9;25:2715. doi: 10.1186/s12889-025-23695-z (PMC12335028; doi:10.1186/s12889-025-23695-z)
Supplement: Supplementary file 18 — Supplementary Material 18. [file 12889_2025_23695_MOESM18_ESM.docx]

**Supplementary Table 12 Correlations of thyroid function and thyroid homeostasis parameters with the all-cause mortality across different CKD progression risk strata based on KDIGO classification.**

|  | **Low risk** | | **Moderate risk** | | **High risk** | | **Very high risk** | |
| --- | --- | --- | --- | --- | --- | --- | --- | --- |
|  | OR (95% CI) | P value | OR (95% CI) | P value | OR (95% CI) | P value | OR (95% CI) | P value |
| **FT3** | 1.02(0.72,1.44) | 0.91 | 0.89(0.66,1.21) | 0.46 | 1.08(1.05,1.11) | <0.0001* | 0.64(0.30,1.34) | 0.23 |
| **FT4** | 1.06(1.02,1.11) | 0.01* | 1.08(1.05,1.11) | <0.0001* | 0.88(0.65,1.19) | 0.41 | 1.03(0.93,1.13) | 0.60 |
| **TSH** | 1.04(1.01,1.06) | <0.0001* | 1.03(1.00,1.05) | 0.03* | 1.03(1.00,1.05) | 0.03* | 0.99(0.94,1.05) | 0.75 |
| **FT3/FT4** | 0.41(0.06,2.78) | 0.36 | 0.17(0.05,0.63) | 0.01* | 0.17(0.05,0.63) | 0.01* | 0.48(0.07,3.10) | 0.44 |
| **TFQI_FT4_** | 1.31(0.90,1.91) | 0.16 | 1.28(1.03,1.61) | 0.03* | 1.28(1.02,1.60) | 0.04* | 0.89(0.49,1.63) | 0.71 |
| **TFQI_FT3_** | 0.85(0.54,1.33) | 0.48 | 0.79(0.61,1.02) | 0.07 | 0.79(0.62,1.01) | 0.06 | 0.73(0.44,1.22) | 0.23 |
| **TT4RI** | 1.01(1.00,1.01) | 0.17 | 1.00(1.00,1.01) | 0.12 | 1.00(1.00,1.01) | 0.13 | 1.00(0.99,1.01) | 0.83 |
| **TT3RI** | 1.01(1.00,1.01) | <0.0001* | 1.01(1.00,1.01) | 0.02* | 1.01(1.00,1.01) | 0.02* | 1.00(0.98,1.01) | 0.74 |
| **TSHI** | 1.03(0.83,1.28) | 0.78 | 1.03(0.92,1.15) | 0.63 | 1.04(0.94,1.16) | 0.46 | 0.93(0.72,1.22) | 0.61 |

Adjusted for age, sex, education level, race, SBP, DBP, BMI, ALT, AST, urine iodine, DM, Hyperlipidemia.

FT3 triiodothyronine, FT4 free thyroxine, TSH thyroid-stimulating hormone, TSHI TSH index, TT4RI thyrotrophic T4 resistance index, TT3RI thyrotrophic T3 resistance index, TFQIFT4, TFQIFT3 thyroid Feedback Quantile-based Index, FT3/FT4 FT3/FT4 ratio

*p≤0.05
