# Supplementary material for: Association between thyroid function and thyroid homeostasis parameters and the prevalence and all-cause and cardiovascular mortality of chronic kidney disease: a population-based study
Source: BMC Public Health. 2025 Aug 9;25:2715. doi: 10.1186/s12889-025-23695-z (PMC12335028; doi:10.1186/s12889-025-23695-z)
Supplement: Supplementary file 19 — Supplementary Material 19. [file 12889_2025_23695_MOESM19_ESM.docx]

**Supplementary Table 13 Correlations of thyroid function and thyroid homeostasis parameters with the cardiovascular mortality across different CKD progression risk strata based on KDIGO classification.**

|  | **Low risk** | | **Moderate risk** | | **High risk** | | | | **Very high risk** | |
| --- | --- | --- | --- | --- | --- | --- | --- | --- | --- | --- |
|  | OR (95% CI) | P value | OR (95% CI) | P value | | OR (95% CI) | P value | OR (95% CI) | | P value |
| **FT3** | 1.03(0.74,1.44) | 0.86 | 0.67(0.46,0.97) | 0.04* | | 0.61(0.43,0.87) | 0.01* | 0.02(0.00, 3.02) | | 0.13 |
| **FT4** | 1.07(1.02,1.11) | 0.002* | 1.03(0.99,1.08) | 0.12 | | 1.04(1.00,1.08) | 0.06 | 0.97(0.87, 1.09) | | 0.64 |
| **TSH** | 1.04(1.01,1.06) | 0.001* | 0.99(0.88,1.11) | 0.86 | | 1.01(0.90,1.13) | 0.89 | 0.62(0.39, 0.98) | | 0.04* |
| **FT3/FT4** | 0.41(0.07,2.62) | 0.35 | 0.37(0.11,1.26) | 0.11 | | 0.26(0.07,0.94) | 0.04* | 0.01(0.00,13.59) | | 0.19 |
| **TFQI_FT4_** | 1.01(1.00,1.01) | 0.19 | 1.09(0.76,1.56) | 0.66 | | 1.26(0.83,1.91) | 0.28 | 0.01(0.00, 1.10) | | 0.06 |
| **TFQI_FT3_** | 0.86(0.56,1.32) | 0.49 | 0.81(0.58,1.14) | 0.22 | | 0.83(0.59,1.17) | 0.29 | 0.02(0.00, 0.91) | | 0.04* |
| TT4RI | 1.01(1.00,1.01) | 0.19 | 1.00(0.99,1.01) | 0.66 | | 1.00(0.99,1.01) | 0.99 | 0.96(0.92, 1.00) | | 0.04* |
| **TT3RI** | 1.01(1.00,1.01) | <0.0001* | 0.99(0.97,1.02) | 0.60 | | 1.00(0.97,1.02) | 0.81 | 0.86(0.77, 0.96) | | 0.01* |
| **TSHI** | 1.02(0.84,1.25) | 0.82 | 1.03(0.84,1.26) | 0.79 | | 1.10(0.87,1.40) | 0.44 | 0.09(0.02, 0.38) | | 0.001* |

Adjusted for age, sex, education level, race, SBP, DBP, BMI, ALT, AST, urine iodine, DM, Hyperlipidemia.

FT3 triiodothyronine, FT4 free thyroxine, TSH thyroid-stimulating hormone, TSHI TSH index, TT4RI thyrotrophic T4 resistance index, TT3RI thyrotrophic T3 resistance index, TFQIFT4, TFQIFT3 thyroid Feedback Quantile-based Index, FT3/FT4 FT3/FT4 ratio

*p≤0.05
